# Insights into Reaction Kinetics in Confined Space: Real Time Observation of Water Formation under a Silica Cover

**DOI:** 10.1021/jacs.1c03197

**Published:** 2021-06-07

**Authors:** Mauricio J. Prieto, Thomas Mullan, Mark Schlutow, Daniel M. Gottlob, Liviu C. Tănase, Dietrich Menzel, Joachim Sauer, Denis Usvyat, Thomas Schmidt, Hans-Joachim Freund

**Affiliations:** † Fritz-Haber Institute of the Max-Planck Society, Faradayweg 4-6, 14195 Berlin, Germany; ‡ Institut für Chemie, Humboldt-Universität zu Berlin, Unter den Linden 6, 10099 Berlin, Germany; § Institut für Mathematik, Freie Universität Berlin, Arnimallee 6, 14195 Berlin, Germany; ∥ Physik-Department E20, Technical University München, James-Franck-Str.1, 85748 Garching, Germany

## Abstract

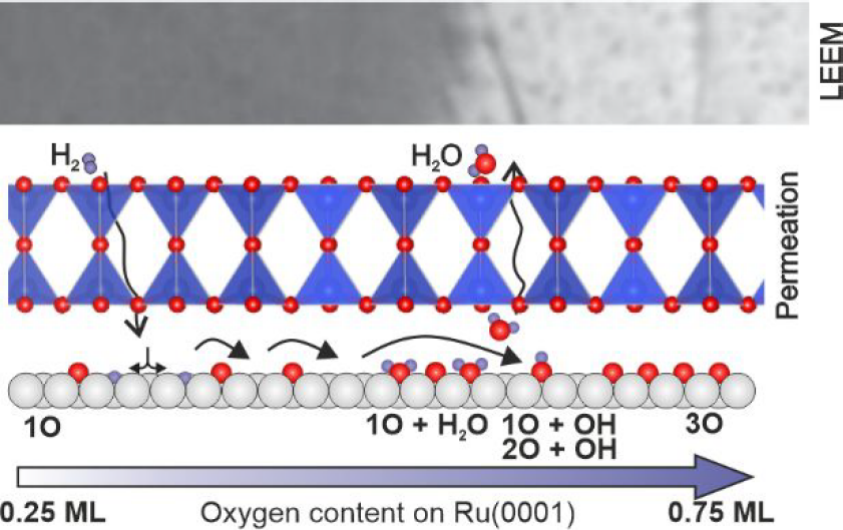

We offer a comprehensive approach to determine how physical confinement can affect the water formation reaction. By using free-standing crystalline SiO_2_ bilayer supported on Ru(0001) as a model system, we studied the water formation reaction under confinement in situ and in real time. Low-energy electron microscopy reveals that the reaction proceeds via the formation of reaction fronts propagating across the Ru(0001) surface. The Arrhenius analyses of the front velocity yield apparent activation energies (*E*_a_^app^) of 0.32 eV for the confined and 0.59 eV for the nonconfined reaction. DFT simulations indicate that the rate-determining step remains unchanged upon confinement, therefore ruling out the widely accepted transition state effect. Additionally, H_2_O accumulation cannot explain the change in *E*_a_^app^ for the confined cases studied because its concentration remains low. Instead, numerical simulations of the proposed kinetic model suggest that the H_2_ adsorption process plays a decisive role in reproducing the Arrhenius plots.

## Introduction

The physical confinement of a chemical reaction has been a topic of great interest in the past decades.^1,2^ The reason for this is the possibility of influencing and even controlling the reaction mechanism by confining not only reactants but also products (see e.g. ref (3)). Multiple arguments have been used to explain the effect of confinement on chemical reactions, and they are based mainly on the fact that when molecules are restricted to small volumes with molecular dimensions, different stabilization mechanisms may come into play. In general, three main effects have been used to describe the effect of confinement. On one side, there is the transition state (TS) effect which describes the stabilization of certain transition structures due to electrostatic and dispersive interactions with the active sites and its surroundings, for example, in zeolites and other nanoporous materials.^4−6^ Furthermore, the size of confinement can introduce steric requirements for molecules participating in the reaction, thus potentially inducing changes in the selectivity of multiple path reactions.^3^ Finally, the dissociative adsorption energy for different molecules can be modified (and potentially tuned) upon confinement, thus altering the catalytic activity on the basis of the Brønsted–Evans–Polanyi (BEP) relation.^7^ In an early review, Csicsery discussed extensively the topic of shape-selective catalysis in zeolite-based materials, identifying three main types of shape selectivity: reactant, product, and transition state selectivity.^8^ In a more recent publication, Clark and co-workers reported a critical analysis of the literature that did not find experimental evidence for true TS selectivity at the time, thus concluding that (hindered) diffusion of the product out of the pores in zeolite materials is responsible for the observed kinetic effects.^9^ Still, this confirms that the task of finding experimental proof of all these concepts in real systems, focusing on the understanding of the fundamental properties of the catalysts, is not trivial, and that requires a combined experimental and theoretical approach.

A tactic that has proven to be quite successful in addressing fundamental questions is the use of model systems that can mimic realistic materials but under controlled conditions.^10−16^ In this sense, thin film silica and aluminosilicate systems supported on transition metal substrates have proven to be suitable candidates as model systems for the study of fundamental properties of zeolites.^17−25^ In the particular case of the SiO_2_ bilayer (BL), two existing polymorphs interacting with its Ru(0001) substrate via van der Waals forces have been reported, namely, crystalline and vitreous. Both polymorphs are held on the metal substrate by van der Waals forces only, thus defining a space where molecules can intercalate. Moreover, they present a well-defined structure that has been unveiled through the combination of techniques such as scanning probe microscopy (STM and AFM),^26−29^ infrared spectroscopy (IRRAS),^17^ low-energy electron diffraction (LEED),^17^ photoemission spectroscopy (XPS),^18,30^ and density functional theory (DFT) simulations.^31,32^ On the atomic scale, both polymorphs consist of corner-sharing SiO_4_ tetrahedra building units with Si–O–Si bridging bonds between the layers, resulting in a ringlike structure that defines channels in the silica framework through which molecules of the right dimensions can diffuse. In the case of the crystalline film, six-member rings (of either O or Si) almost exclusively form the bilayer structure, with the exception of the 48, 75, 558, and 5775 ring arrangements found at domain boundaries.^33^ On the other hand, the vitreous BL exhibits a broad distribution of ring sizes resembling the structure of three-dimensional glass.^29,34,35^ It is the presence of these channels acting like pores in both silica polymorphs that makes the system interesting for intercalation and reactivity studies. For instance, it has been reported that O_2_, D_2_, and H_2_ can be intercalated in the space defined between the SiO_2_ and the Ru(0001) substrate,^36,37^ with the activation energy to diffuse through the film depending on the ring/molecule size.^38,39^ In this sense, the space confined between the SiO_2_ BL and the ruthenium support can be used as a nanoreactor for chemical reactions by using the intercalated gases as reactants. Moreover, because of its porous nature, the silica BL film can act as a molecular sieve in complex reactions leading to products having different molecular sizes.

In the present publication we report a broad study of the effects of physical confinement on the kinetics of the water formation reaction (WFR) using a silica bilayer supported on ruthenium as a model system. The combination of an experimental approach with a theoretical description based on DFT and microkinetic simulations allows us to address how physical confinement of the reactants and the product affects the observed kinetics, bridging the different scales from an atomistic to a mesoscopic description of the reaction. For instance, we find that the rate of hydroxyls formation on the ruthenium surface remains unchanged. Modeling of the kinetic equations shows that under our experimental conditions water entrapment cannot solely explain the observed sluggishness of the reaction rate under confinement. Even though the water concentration increases in comparison with the uncovered Ru case, it remains too low for it to strongly affect the kinetics. Instead, we find that it is rather the effective rate of H adsorption in the first step of the mechanism that is strongly affected by the presence of the silica film, thus making the SiO_2_|Ru(0001) system an interesting model system for study. This unique approach allows us to establish general correlations and, more importantly, to identify the affected steps in the reaction mechanism.

In a previous communication we have reported the preliminary study of the WFR confined under a vitreous SiO_2_ bilayer and its Ru(0001) support, with a big influence on the apparent activation energy of the reaction upon confinement.^40^ By means of low-energy electron microscopy (LEEM), we determined that the water formation reaction proceeds with the formation of reaction fronts that propagate across the surface with variable speeds depending on the sample temperature. The existence of a reaction front is explained by the coexistence of areas having different oxygen coverage (θ_O_ = 0.75 and 0.25 ML for unreacted and reacted areas, respectively) on the Ru surface, namely, O-rich and O-poor. It is only on the O-poor side that the dissociative adsorption of H_2_ can occur because of its two-free-sites requirement. Subsequently, the adsorbed H has to diffuse to the O-rich area where it reacts with O. Because the relatively high oxygen coverage at the initial stages of the reaction prevents the dissociative adsorption of H_2_ due to the two neighboring site requirement, we concluded that the starting point of the front propagation must be located at a defect or vacancy in the 3O layer resulting from the film preparation step.^40^

Since our study of the vitreous SiO_2_ bilayer, new studies have been reported on this topic by using integral techniques and attempting an atomistic description of the process under two-dimensional silica or aluminosilicates.^41,42^ However, to the best of our knowledge, a detailed study of the kinetic aspects of confinement aiming at understanding the distribution of species across the reaction fronts and the differences in the apparent activation energies is still missing. For this reason, we propose the use of a purely crystalline SiO_2_ BL film in the study of the WFR in confinement, since a more structurally defined bilayer film can provide a better assessment and correlations with properties derived from DFT simulations and, ultimately, from the modeling of the reaction mechanism.

**Figure 1:**
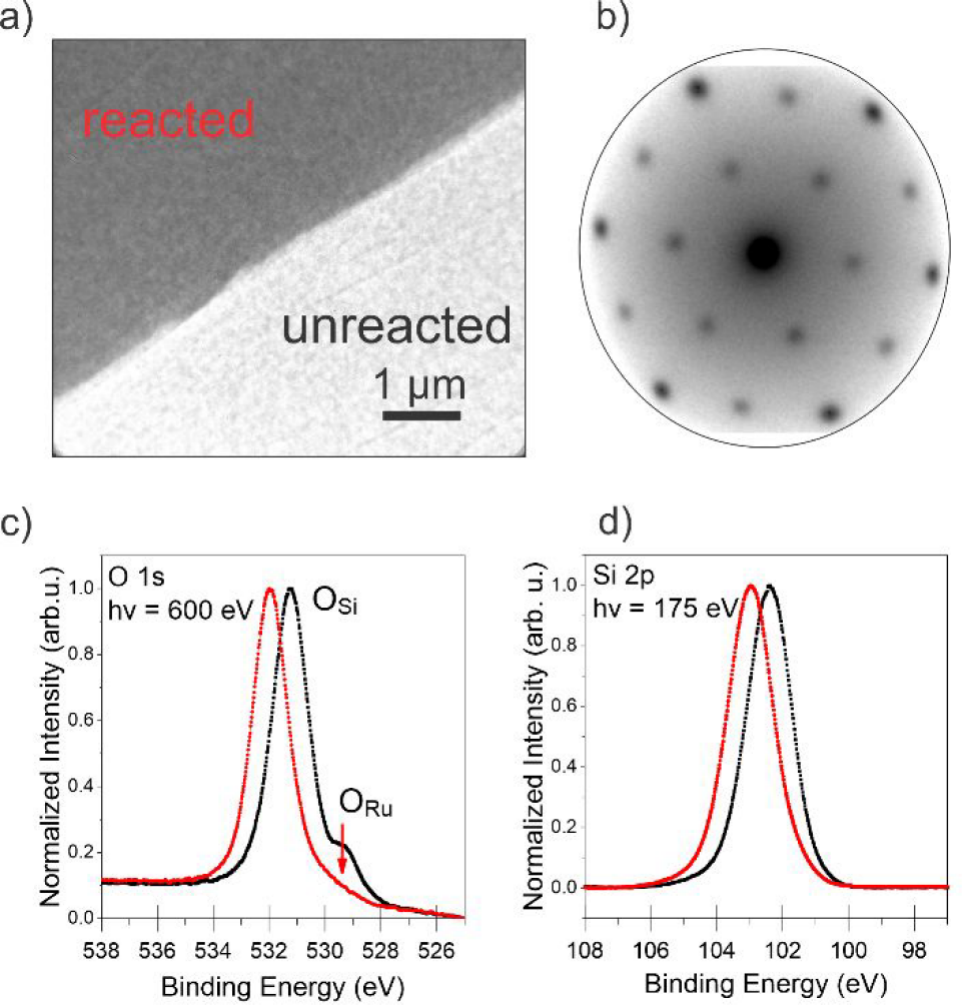
(a) LEEM snapshot showing the reaction front under reaction conditions; the O-rich and O-poor areas are labeled as unreacted (black) and reacted (red), respectively; the electron energy is 10 eV. (b) LEED pattern at an electron kinetic energy of 42 eV showing the characteristic (2 × 2) spots of the SiO_2_ BL on Ru(0001) measured on the reacted sample. However, except for the intensity, the patterns of the reacted and unreacted surface do not differ regarding number, position, and broadening of the spots. A comparison of the patterns at different stages can be seen in Figure S10. (c, d) Local O 1s and Si 2p photoemission spectra collected on both sides of the reaction front, as indicated.

Figure 1 summarizes our findings with the crystalline SiO_2_ BL/nO/Ru(0001) system. It is evident from the LEEM snapshot of Figure 1a that the WFR proceeds in a similar fashion as for its vitreous counterpart,^40^ with the formation of propagating fronts when the sample is annealed in 1 × 10^–6^ mbar of H_2_. A time series of LEEM images collected during reaction and movies showing the progression of the reaction are provided in the Supporting Information. As in the case of the vitreous silica film, no preferential orientation for the front movement is observed. A thorough characterization of the different areas (bright and dark) was performed in static conditions, once the front propagation was stopped and stabilized by rapidly cooling the sample. Our results show that the structure of the silica film is not affected by the reaction front, as indicated by the characteristic (2 × 2) spots in the LEED pattern in Figure 1b and by XPS data. The local XPS (μ-XPS) data in Figure 1c,d show that the chemical state of the silica bilayer is not compromised during the reaction, as suggested by the comparison of the Si 2p line shape as well as that of the main component under the O 1s line.

However, the consumption of interfacial oxygen (O_Ru_) can be determined by examining the intensity of the small component at lower binding energies in the O 1s spectra (red arrow). Moreover, the binding energy shift of both the Si 2p and the O 1s main components is observed as a consequence of the removal of the interface dipole when removing O_Ru_.^18,30^ On the other hand, the possibility of O_Ru_ removal due to thermal desorption is discarded based on the fact that the onset temperature reported for O_2_ desorption is well above (∼1000 K)^43^ the reaction temperatures used in our experiments (400–675 K). All these findings confirm that the WFR proceeds in a similar fashion under a crystalline and a vitreous phase, with the origin of intensity change across the front being the variation of the O_Ru_ concentration. Moreover, because the changes and fingerprints observed with both polymorphs under reaction conditions are similar, we conclude that the reaction proceeds following the same mechanism in both cases.

## Results and Discussion

The analysis of the temperature dependence of the propagation velocity of the front shown in Figure 1a was performed to determine the apparent activation energy (*E*_a_^app^) for the reaction with and without confinement. It is important to mention at this point that when the 3O/Ru(0001) surface (without the SiO_2_ cover) is submitted to the same reaction condition, a reaction front having similar characteristics as those described above is observed, thus providing a point of comparison to address the effect of confinement on the water formation reaction. Because the front velocity (*v*_front_) can be directly related to the velocity at which O_Ru_ is consumed by reacting with H_2_, it is an easily accessible parameter in LEEM and a suitable choice for the indirect assessment of the reaction rate. A detailed description on how *v*_front_ can be obtained from the variation of the LEEM image intensity during the propagation of the front can be found in ref (40).

**Figure 2:**
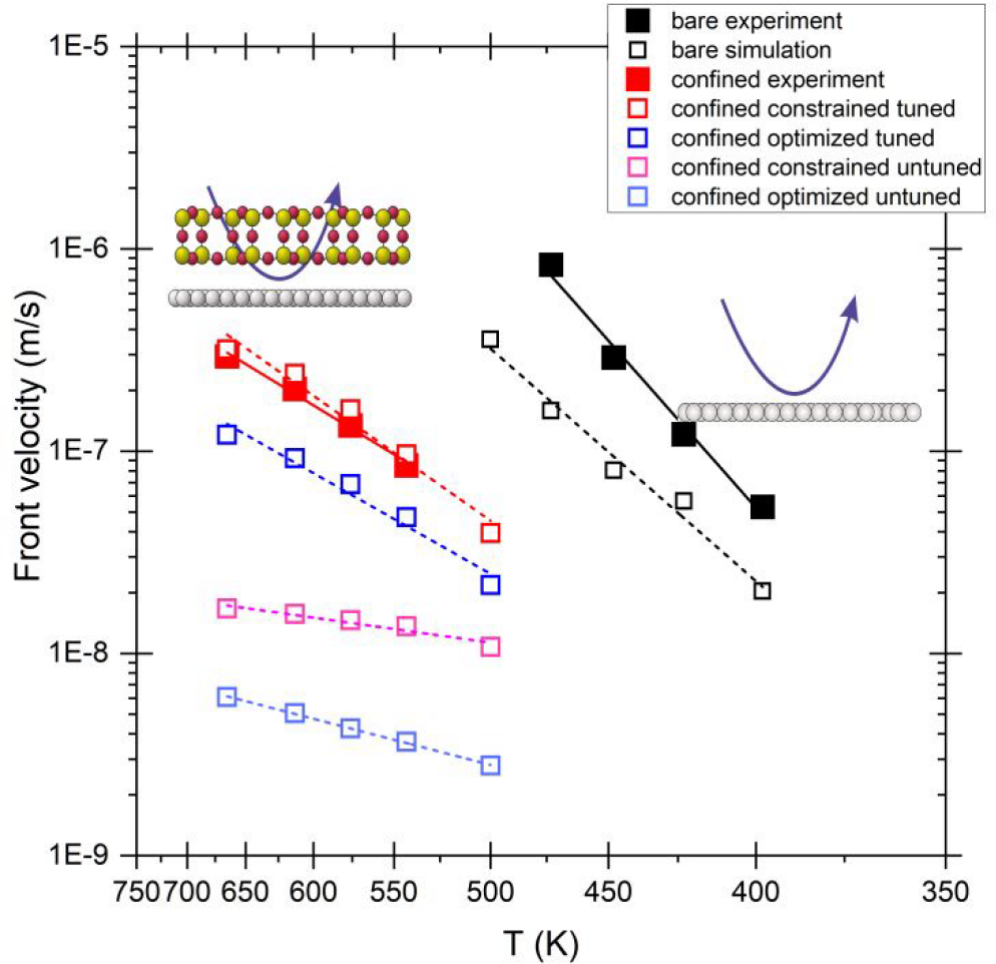
Experimental and theoretically derived Arrhenius plots of the reaction front velocity as a function of temperature obtained for the nonconfined and confined reactions and uncovered Ru(0001), as indicated. Simulated curves exhibited correspond to the data set showing the best fits. Untuned and tuned correspond to scenarios used in the numerical simulations of the kinetic modeling where the H_2_ adsorption step is purely defined by DFT or tuned from those values, respectively. See the text for more details.

Figure 2 shows the Arrhenius plots constructed from the temperature dependent front velocities for both uncovered and covered Ru(0001) surfaces as well as for different theoretical scenarios considered in our studies (*vide infra* for more details). Our determinations show that confining the reaction under a crystalline silica bilayer yields a decrease in the *E*_a_^app^ for the propagation of the reaction front from 0.59 to 0.32 eV, with the value for the uncovered case being in excellent agreement with data reported by integral methods.^44,45^ The reduction by half of the apparent activation energy suggests that the reaction becomes diffusion controlled under confinement.^46,47^ It is important to note that when comparing the behavior with that of the vitreous SiO_2_ BL system, the *E*_a_^app^ obtained are within the level of accuracy of our measurements (0.32 vs 0.27 eV). However, a clear difference in the front propagation velocities is observed, with that of the vitreous film moving on average 18% faster than under the crystalline film. A plausible explanation for this observation will be given in the following paragraphs once the kinetic model is properly introduced and discussed.

Even though it is clear from the Arrhenius plots of Figure 2 that confinement under the SiO_2_ crystalline bilayer has a strong influence on the energetics of the water formation reaction catalyzed by the Ru(0001) surface, it is not a trivial task to identify which step in the whole reaction mechanism is indeed affected. We propose the following reaction steps as a generalized mechanism for the reaction, regardless of the existence of confinement.
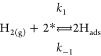
1
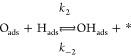
2
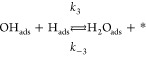
3
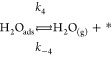
4
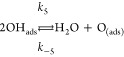
5where ∗ represents a free active site on the surface and *k*_*i*_ and *k*_–*i*_ represent the kinetic constant for each reaction step in the forward and backward directions, respectively. Steps 1–4 have been identified previously in the case of H_2_O formation on Ru(0001) (see refs (44 and 45) and references therein). The authors demonstrated that step 2 must be the rate-limiting step for the water formation on bare Ru, with all the subsequent steps being considerably faster. The water desorption in step 4 is particularly fast, provided that the reaction is performed at temperatures at which the thermal desorption from Ru(0001) is completed (above ∼220 K).^48^Equation 5 has been included in our analyses based on evidence presented by Ertl and co-workers on Pt(111), where the comproportionation/disproportionation paths become relevant at low reaction temperatures (*T* < 200 K).^49^ Although the likelihood of reaction 5 was discarded in previous studies on bare Ru(0001), it seems reasonable to include it due to the anticipated higher temporary water coverage under the silica films.

On the basis of the mechanism presented above, we performed DFT simulations with the aim of obtaining the activation energies for the different elementary steps presented in eqs 1–5 on both covered and uncovered Ru(0001). Two different aspects are crucial to determine the boundary conditions for our calculations. First, as discussed before, the reason for the existence of a front can be tracked down to the fact that molecular hydrogen needs two adjacent sites to adsorb on Ru(0001), and the oxygen coverage is not homogeneous across the active surface.

Second, only two oxygen adatoms within the 3O layer can be removed by H_2_ at the reaction temperatures used in our experiments, thus leaving one unreacted adatom still adsorbed in the (2 × 2) unit cell. According to previous studies, temperatures above 970 K are needed to completely remove all adsorbed oxygen atoms in the H_2_ pressure range used in our studies.^44^

Considering these two points, the situation represented in the snapshot of Figure 1a can be rationalized in the following way. The bright area of the snapshot corresponds to an area having an oxygen coverage corresponding to the 3O phase (0.75 ML). On the other hand, the dark area far away from the front region must correspond to the fully reacted surface, where a coverage equivalent to the 1O phase is expected (0.25 ML). It is in the region at the vicinity of the front where the WFR occurs, and also, the transition from the 1O to the 3O phase is expected. Thus, in some areas of the front an intermediate (and continuously changing) oxygen coverage is expected (0.25 < θ < 0.75). Interestingly, previous work identified 0.37 ML of O_Ru_ as the most reactive phase for the reaction conducted at much lower temperatures compared to our experiments.^44,45^ On the basis of all this, we assume that H_2_ can only dissociate through eq 1 on the dark side of the front where a lower oxygen content is available; it must then diffuse across the surface and reach the front to react further through the steps 2 and 3 to form water. For this reason, our DFT calculations use the 1O layer for the adsorption process represented by eq 1 and the 2O layer for all processes represented by eqs 2–5 for the confined and nonconfined cases. Moreover, we find that the energy barriers for the main reaction steps are noticeably higher than the secondary ones (diffusion through the silica film), so for the hydrogen adsorption and desorption process, the highest energy point was considered as the transition state, which was then used to calculate the respective Gibbs free energies. More details can be found in section S4 of the Supporting Information.

**Figure 3:**
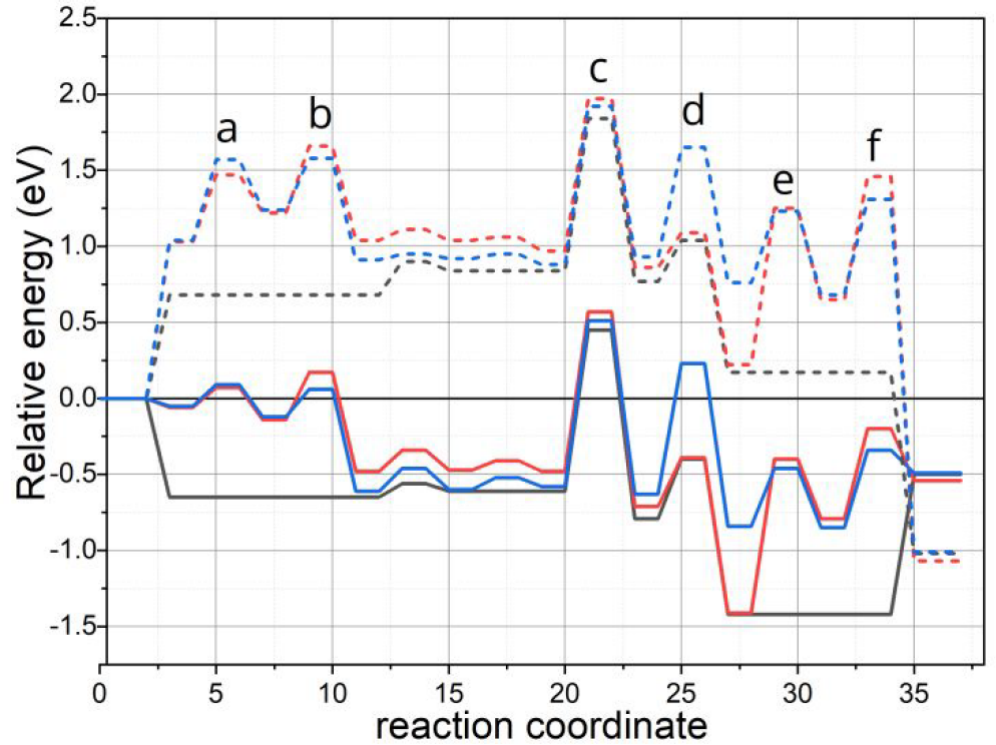
Energy diagrams obtained from DFT simulations for the water formation reaction without (black) and with (red: optimized SiO_2_ BL; blue: constrained SiO_2_ BL) confinement according to the mechanism described in eqs 1–4. Solid lines correspond to pure electronic energy values, and dashed lines correspond to Gibbs free energy values calculated at 500 K, taking hydrogen in the gas phase as reference. The first two states in the reaction coordinate (H_2(g)_ and TS_H_2_) correspond to processes occurring on a 1O-Ru(0001) surface, while the following ones correspond to those starting from a 2O-Ru(0001) phase. Letters in the inset correspond to the H_2_ adsorption (a, b), OH formation (c), H_2_O formation (d), and H_2_O desorption (e, f).

Figure 3 shows the pure electronic energy diagram and the Gibbs free energies calculated at 500 K for the water formation on bare and SiO_2_ covered Ru(0001), as indicated, starting with H_2_ in the gas phase on the left, followed by dissociative H_2_ adsorption, OH and subsequent H_2_O formation, and finally H_2_O desorption. Because of the lack of experimental evidence, the atomic position of the silica bilayer relative to the Ru(0001) support must be arbitrarily chosen during simulations. However, its relative position can have a strong effect in some of the elementary steps of the reaction, especially those that involve the interaction of reactant/product with the film, that is, H_2_ adsorption and H_2_O desorption. For this reason, two extreme scenarios (optimized and constrained BL) were considered.

It is clear from the energy diagram that among the two scenarios considered under confinement the rate-determining step (rds) remains virtually unchanged and also unchanged from that of the nonconfined situation. Therefore, the rds corresponds in all cases to the formation of OH_ads_ represented by eq 2 and involving transition state (c) (TS-c). Details on the evolution of the structures along the path described by eq 2 can be found in the Supporting Information (see Figure S11). On the basis of these results, we conclude that the changes introduced by confinement are not related to the traditional transition state effects observed in zeolites, where stabilization of the transition complex is responsible for the changes in reactivity.^4,6,50^

A close inspection of the energy diagrams reveals that the presence of the SiO_2_ introduces additional transition states that were not present on the open Ru(0001) surface and that their relative height strongly depends on the relative position of the silica bilayer. For instance, the first two transition states (a, b) are related to the permeation of H_2_ molecules in the gas phase through both layers of the SiO_2_ film before dissociating on the Ru(0001) surface. The fact that the presence of the SiO_2_ introduces additional steps due to its permeability is well-documented in the literature for the SiO_2_ system.^36−39^ However, the implications that these additional steps may have on the reaction kinetics is a new aspect that, to the best of our knowledge, has never been addressed experimentally before.

**Figure 4:**
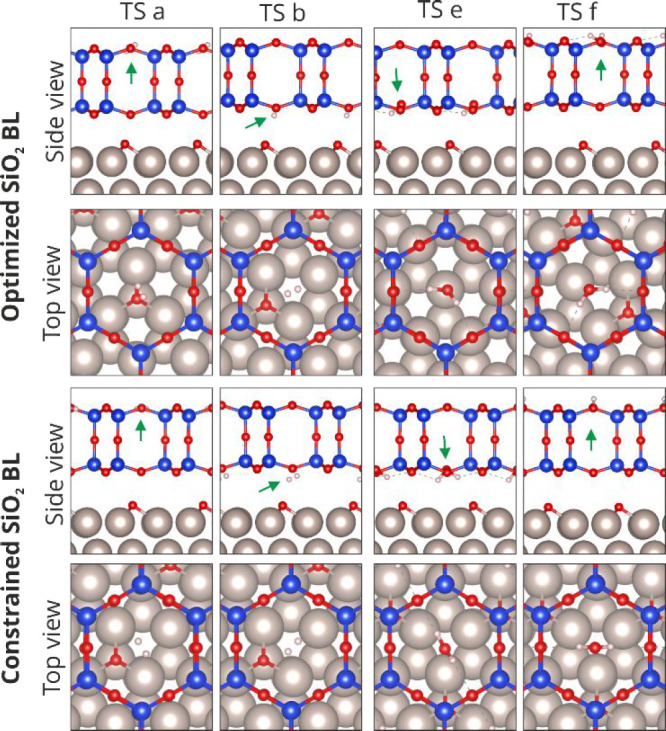
Structures of the transition state involved in the hydrogen adsorption (TS a, TS b) and water desorption (TS e, TS f) steps under confinement. Green arrows indicate the position of H_2_ and H_2_O molecules.

On the other hand, the last step of the reaction is also affected by the presence of the silica film. For instance, in a pathway analogous to the H_2_ intercalation before adsorption, the process represented by eq 4 requires the migration of H_2_O molecules through the silica film after desorption from the Ru surface. All new TS structures introduced by the mere presence of the silica lid are presented in Figure 4. The proximity of both molecules to the silica film confirms that the origin of the additional barriers lies in the interaction of the molecules with the thin oxide film rather than with the Ru(0001) substrate. This finding is in good agreement with the fact that comparable pore diameter in the film (0.47 nm for six-member rings^31^) and molecular dimensions (H_2_O: 0.282 nm;^51^ H_2_: 0.210 nm^52^) are expected. It is important to point out that TS a,f and TS b,e correspond to stages involving the interaction of the corresponding molecules with the top and bottom layers of the BL, respectively. Interestingly, contrary to what was reported for other molecules confined under 2D materials,^7^ we have not identified changes in the adsorption site preference/energies of all intermediates along the reaction for the confined and nonconfined cases. For comparison, we provide the structure files of all transition and intermediate states in the Supporting Information.

The last prominent difference in the energy diagram is observed in the transition state associated with eq 3 (TS d). Here, it is clear that even though the transition state for the formation of water through the final H addition step to OH is only slightly changed in the case of the fully optimized silica BL, the situation for the constrained BL is rather different. For instance, fixing the position of the silica lid has a strong influence in the energetics of the transition state, where much higher activation energy is anticipated for this elementary step (by 0.72 eV at 500 K).

Close inspection of the activated complex structures reveals that in the reaction on uncovered and optimized BL covered ruthenium the bond distances in the H–O–H species are virtually identical (*d*_H–O_: 1.54 Å; *d*_H–O_: 0.98 Å); however, the bond angle differs slightly (bare: 101.6°; optimized BL: 103.7°). On the other hand, even though bond distances change slightly when the lateral position of the BL is constrained (*d*_H–O_: 1.49 Å; *d*_H–O_: 0.98 Å), the bond angle is significantly affected rendering a value of 109.6°, much higher than the expected value for a fully formed H_2_O molecule (104.5°). The differences in the bond angles of the activated complex can be rationalized by the fact that in the first two scenarios the reaction path leads to the formation of the activated complex in a top position at the center of a six-member ring, thus optimizing the interaction with the bilayer. Oppositely, the off-centered configuration in the case of the constrained silica film may offer a less favorable interaction between the intermediate species and the film.

**Figure 5:**
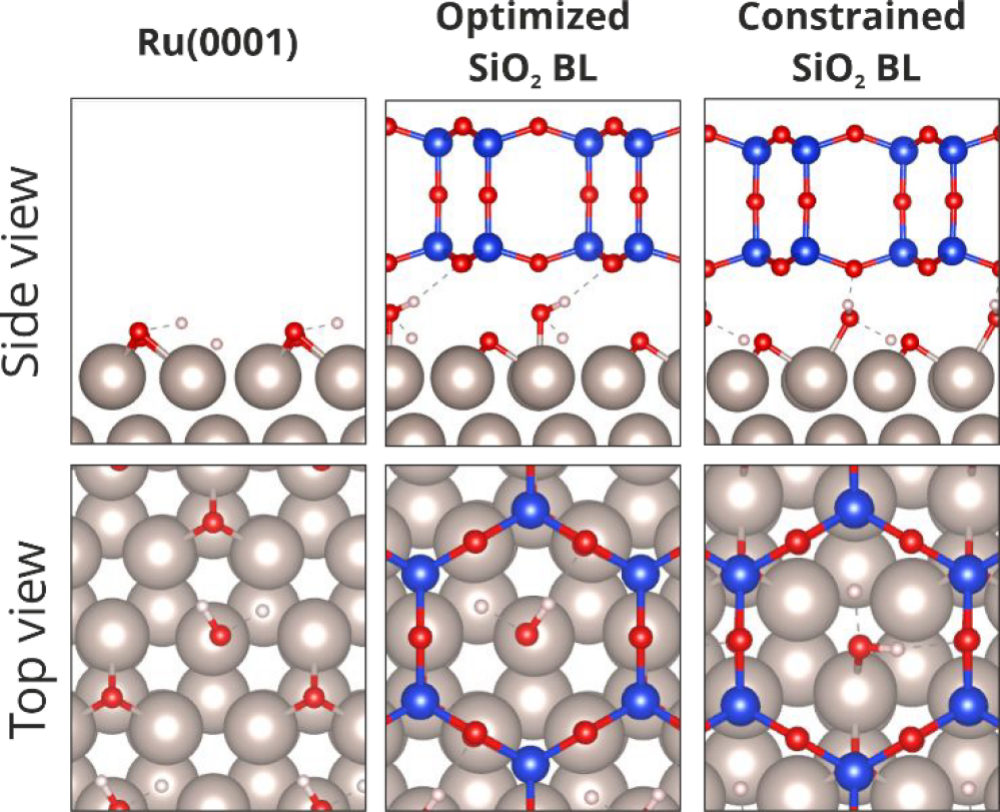
Structures of the transition state (TS d) involved in the final step for the water formation process prior to its desorption.

To evaluate the kinetic aspects of the global reaction, the kinetic constants *k*_*i*_ for each elementary step, as well as the diffusion constant *D*_H_ for H on Ru(0001), were calculated for the confined and nonconfined WFR (see sections S7–S9 in the Supporting Information). The values obtained are listed in Table 1. The complete set of kinetic constants at all studied temperatures can be found in the Supporting Information.

**Table 1:** Kinetic Constants (*k*_*i*_) Obtained Directly from the DFT Calculations (Untuned) for the Different Reaction Steps Presented in Eqs 1–4 as Well as That for H Diffusion on Ru(0001) and Two SiO_2_/Ru(0001) Scenarios^a^

*k*_i_	nonconfined	confined optimized BL	confined constrained BL
*k*_1_ [m^2^/s]	3 × 10^–15^	4 × 10^–23^	4 × 10^–22^
*k*_–1_ [m^2^/s]	7 × 10^–7^	3 × 10^–13^	3 × 10^–13^
*k*_2_ [m^2^/s]	2 × 10^–16^	2 × 10^–16^	1 × 10^–16^
*k*_3_ [m^2^/s]	5 × 10^–9^	1 × 10^–8^	1 × 10^–13^
*k*_4_ [1/s]	1 × 10^13^	4	2 × 10^8^
*D*_H_ [m^2^/s]	5.6 × 10^–9^	1.9 × 10^–9^	1.9 × 10^–9^

a All constants shown correspond to a reaction temperature of 500 K.

As can be clearly seen from the table, the values of *k*_1_, *k*_–1_, and *k*_4_ reflect the points addressed before; that is, the most affected steps upon confinement are the adsorption of H_2_ and the desorption of H_2_O. It is worth mentioning at this point that in the case of the confined reaction, since the desorption of water molecules involves overcoming two transition states, the kinetic constants reported correspond to effective constants. The methodology used for their calculation can be found in the Supporting Information. On the other hand, the lack of TS in water desorption for the nonconfined reaction translates into high values for this system. The high *k*_4_ values in this case can be rationalized in terms of extremely short residence times of H_2_O molecules on Ru(0001) after their formation by eq 4, in agreement with previous reports by other authors.^53,54^

The following set of differential equations was built to account for the spatiotemporal dependence of the surface concentration *n*_*i*_ = *n*_*i*_(*x*,*t*) of all species involved in the reaction mechanism, based on the kinetic model presented in eqs 1–4.
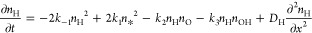
6
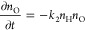
7
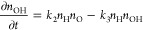
8
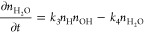
9
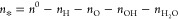
10With *n*^0^ being the density of possible free sites. In a (2 × 2) unit cell there are three possible free sites for adsorption since one is permanently occupied with an O atom that is not removed under our experimental conditions. Therefore,  m^–2^ with *A*_*cell*_ = 2.53 × 10^–19^ m^2^ being the area of a (2 × 2) unit cell.

Because the disproportionation/comproportionation paths represented by eq 5 constitute a branching of the main reaction mechanism (eqs 1–4) that could affect the front speed, it seems relevant to discuss its likelihood under the present conditions. For instance, on bare Pt(111) Ertl and co-workers have proven that the paths exist at low reaction temperatures, where water desorption is strongly hindered.^55^ In the case of bare Ru(0001) these paths have been discarded based on the fact that the presence of OH_ads_ could not be detected with XPS nor HREELS, thus suggesting its concentration must be very low.^44^ However, Wang et al.^42^ recently reported in their study at higher H_2_ pressures the possibility of water entrapment under the silica film, driven mainly by the relatively low activation energy for the path represented in eq 5 and the supposedly high water concentration at the interface.

Our DFT simulations show that even though the energy barrier for the disproportionation is quite low, the activation energies for the comproportionation paths are considerably higher. The electronic minimum-energy paths for both reactions can be found in section S5 of the Supporting Information (Figure S5). More importantly, our results indicate that upon confinement the energy barriers are strongly influenced by the relative position of the silica film, especially in the case of comproportionation. Nonetheless, all *E*_a_ of the steps involved are comparable to (or even lower than) that of the step represented by eq 3 (see energy diagram in Figure 3 for comparison). Our results then suggest that for these paths to contribute to the overall reaction rate, and therefore to the front speed, the concentration of the involved species must be high enough.

From the differential equation system provided in eqs 6–10 the amount of water molecules formed can be estimated from  for all cases. The maximum coverage values calculated are 3.9 × 10^–15^, 1.7 × 10^–3^, and 5.4 × 10^–11^ ML for the nonconfined, confined optimized, and constrained BL, respectively. On the basis of this argument, we conclude that the contribution of the disproportionation path to the global reaction is negligible. Also, on the basis of calculations derived from our kinetic model, we estimate much lower *k*_4_ values (at least 2 orders of magnitude) are necessary to reach water coverages under the silica film for the comproportionation path to become relevant.

From the calculated kinetic constants for H_2_O desorption (*k*_4_ in Table 1) one can infer that the residence time of H_2_O molecules on Ru is ∼0.25 s in the confined optimized scenario, with much shorter times in the cases of the constrained BL and nonconfined reaction (5 × 10^–9^ and 10^–13^ s, respectively). By considering the H_2_O diffusion coefficients obtained in our DFT simulations (*D*_H_2_O_ = 3.6 × 10^–16^–1.0 × 10^–14^ m^2^ s^–1^ at 500–615 K), we estimate average traveling distances of 3 × 10^–3^ nm (optimized BL) and 20 nm (constrained BL) for a H_2_O molecule in confinement, before desorbing. These values indicate that water molecules formed will desorb from areas within the reactive front. Moreover, the dimensions cited above are within the range reported for the existence of domain boundaries in the crystalline silica bilayer polymorph (5–15 nm), where eight-member rings offering less resistance for H_2_O permeation can be present.^33,56^ An important point to be mentioned is that in the comproportionation path the water molecule formed from the 2O reactive phase must react with the remaining O adsorbed on Ru(0001) (formally named 1O). However, the removal of this remaining—rather unreactive—O adatom from the Ru surface was not observed in our experiments since it requires temperatures higher than those used in our experiments in the 10^–6^ mbar H_2_ pressure range, in agreement with previous reports.^44^

On the other hand, on the basis of the same concentration arguments, we discard the possibility of a strong contribution to the global reaction by the disproportionation path. The maximum attainable OH coverage nearby the reaction front and under reaction conditions can be estimated from eq 7 as  ≃ 3 × 10^–8^ ML on the bare surface, in agreement with the extremely short times we determined for H_2_O_ads_ on bare Ru. Estimations of θ_OH_^max^ in both confined cases yield values of 1.5 × 10^–8^ and 7.5 × 10^–4^ ML for the optimized and constrained BL. Of course, local diffusion of two OH groups into the same unit cell could increase the contribution of the path to the reaction rate, but activation energies for OH diffusion have been found to be considerably high (see section S7 of the Supporting Information for a detailed description of this step).

Our differential equation system considers only the diffusion of H_ads_ in the last term of eq 6, thus disregarding the H_2_O diffusion on Ru(0001) as a relevant process for the front propagation. The reason for this assumption is twofold: First, in the case of the nonconfined situation, H_2_O molecules instantly desorb at the temperatures at which the reaction runs in our experiments, making the average lifetime of adsorbed water extremely short. Second, even though the water desorption rate is considerably lowered by the presence of the silica bilayer in the confined reaction, our DFT simulations suggest that the electronic energy barriers for different migration paths on Ru are rather high (∼0.8 eV) for a site hopping mechanism, mainly because the oxygen atom in the water molecule repels the oxygen atom in the bottom layer of the silica film. It is also worth mentioning that in the case of H an alternative diffusion path by hydrogen hopping over neighboring surface O atoms was discarded due to the higher activation energy associated with it (∼0.7 eV) and because the major contribution to H adsorption and diffusion comes from regions of the sample where a 1O coverage is expected. Thus, the formation of an OH group after the dissociative adsorption of H_2_ (within the 1O region) is the first step of the hopping mechanism, with a rather high activation energy (∼1.3 eV). On the other hand, neighboring O adatoms providing consecutive hopping sites are missing in this region, given that only one oxygen atom per unit cell is available in this phase.

The numerical simulations of the kinetic model presented above were performed by using two approaches. First, the kinetic parameters directly obtained from DFT at variable temperature were used as starting point (see Table S1). Second, the values of the kinetic constants representing the adsorption/desorption of H_2_ (*k*_1_/*k*_–1_) were carefully adjusted (“tuned”; see the summary in Table S2 for a comparison). By computing the front velocities at different temperatures in both approaches, we were able to reconstruct the Arrhenius plots entirely derived from theoretical values and compare them with those experimentally determined. All Arrhenius plots resulting from these simulations are shown in Figure 2. From the plots we conclude that the first step in the reaction mechanism, that is, H_2_ dissociative adsorption/desorption, has a strong influence in both the temperature dependence and the magnitude of the front velocity. The effect becomes evident when cases 1 and 2 are compared for the confined reaction.

For instance, the Arrhenius trend can be reproduced with a reasonable degree of agreement by tuning the hydrogen adsorption step. The “tuned” case considers the possibility that the values for the constants *k*_1_ and *k*_–1_ are much higher (by a factor of 1000) than those directly obtained from DFT calculations. The fact that the ratio *k*_1_/*k*_–1_ is preserved (both constants are equally scaled) indicates that the average attainable coverage of H_ads_ remains unchanged, although the equilibrium condition is achieved much faster in comparison with the untuned situation. In the case of the nonconfined reaction, the effect observed is not as strong as for the confined reaction, thus suggesting that H_ads_, necessary for the ignition of the cascade of elementary steps, is readily available in all conditions. Interestingly, even though the crystalline silica bilayer is porous enough to allow the H_2_ molecules to permeate it and reach the Ru surface, it is the H-adsorption step that becomes affected by the presence of the silica lid. Moreover, this molecular step is the one that considerably affects the observed apparent activation energies rather than the water desorption/readsorption as reported in ref (42), at least in the pressure range of our study. It is worth mentioning that the same approach used for *k*_1_ and *k*_–1_ was applied for *k*_3_ and *k*_4_. Interestingly, varying the kinetic constants for water formation and desorption does not affect the front speed in our simulations, thus indicating that once OH is formed through eq 2 none of the following steps determine the reaction rate, provided that all following steps are faster than the formation of OH.

**Figure 6:**
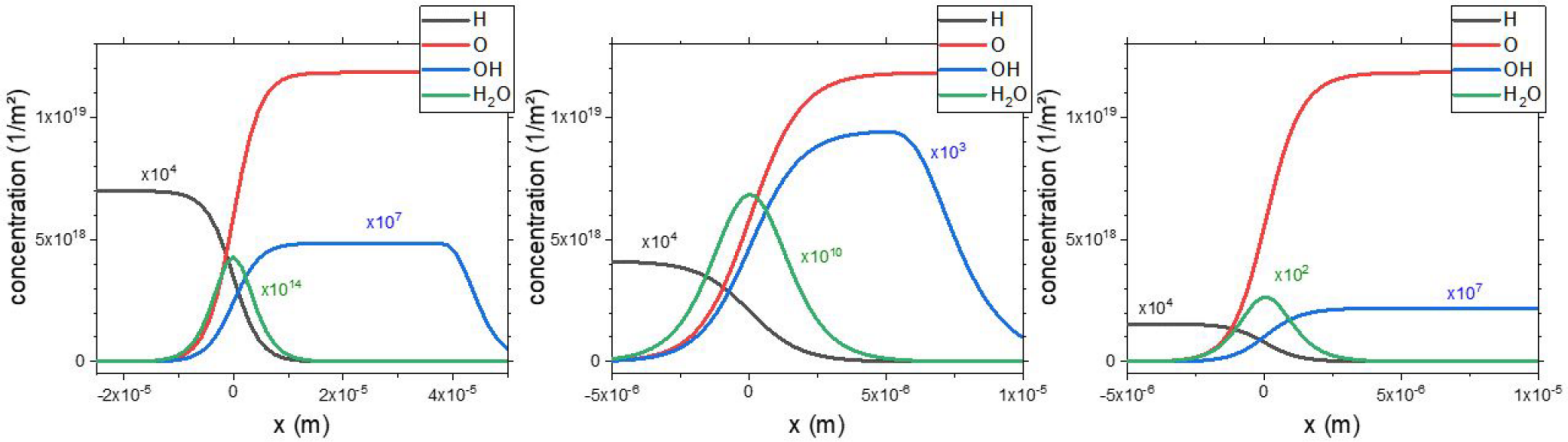
Concentration profiles of H_ads_, O_ads_, OH_ads_, and H_2_O_ads_ for the water formation reaction obtained from the numerical simulations on (a) bare Ru(0001), (b) in confinement under a constrained SiO_2_ crystalline bilayer, and (c) in confinement under an optimized SiO_2_ crystalline bilayer. All concentration profiles correspond to the simulations exhibiting the best fits in the Arrhenius plots with the experimental profiles. Profiles calculated at 500 K by using *k* values for untuned and tuned cases for the nonconfined and confined optimized and constrained BL scenarios, respectively. Note that the scaling of the *y*-axis is identical, while in the case of the *x*-axis there is a difference between confined and nonconfined situations. The corresponding set of kinetic constants for each case can be seen in Tables S1 and S2.

Figure 6 presents the spatiotemporal surface concentration profiles obtained for all species involved in the WFR (H_ads_, O_ads_, OH_ads_, and H_2_O_ads_) for the three different scenarios. These results show that the proposed kinetic model can successfully explain the formation of reaction fronts on the ruthenium surface as the reaction proceeds both on the open surface and under confinement. More importantly, the reaction velocities obtained from our mathematical model reproduces the trend observed in the experimental values; that is, the reaction front moves slower under confinement.

From the concentration profiles, we conclude that the active area during the reaction is the region in the vicinity of the moving front, where the concentrations of OH and H_2_O are maximized. This means that even though there is a wide area where the two-site requirement can be fulfilled for H_2_ adsorption (dark side of the front containing the 1O/Ru(0001) phase), only those H adatoms that are close to the border of the front will be able to propagate the reaction front, on the condition that their diffusion is not hindered and any free site is fast filled up with hydrogen. Particularly interesting is the profile observed for the OH species. For instance, it is clear from the concentration profiles that the presence of OH can extend over a broad area, in confined and nonconfined cases, with a somewhat higher concentration for the constrained BL. These findings suggest that H adatoms may be able to penetrate the region where the unreacted 3O layer exists, a somewhat unexpected outcome considering that on the unreacted side of the front (3O-Ru(0001)) only one free site is available for H_ads_. It is important to point out that this behavior could result from a limitation of our model, where the diffusion coefficient has been considered equivalent on areas having different oxygen coverage. In this sense, it becomes relevant to address additional diffusion mechanisms for atomic H through different oxygen containing areas on Ru(0001) (i.e., concentration-dependent diffusion coefficient), an issue that has not been considered so far and constitutes the core of future studies.

On the basis of all the arguments exposed so far, we offer the following model to describe the effect of the silica bilayer on the kinetics of the WFR. Our study shows that two steps are important to describe the experimental front speed values. These are (i) the dissociative adsorption of H_2_ (involving *k*_1_ and *k*_–1_) and (ii) the reaction of H_ads_ and O_ads_ to form OH_ads_ in the second elementary step (involving *k*_2_). All following processes in the reaction mechanism (*k*_3_ and *k*_4_) are faster and do not limit the reaction speed, even under confinement. When the hydrogen adsorption process is fast enough (as in the nonconfined reaction), plenty of hydrogen is available on the ruthenium surface to propagate the reaction front. Under these circumstances, the reaction frequency is determined solely by the formation of OH on ruthenium, a process represented by *k*_2_. However, even though the presence of the silica bilayer does not affect considerably the average hydrogen coverage on Ru under equivalent experimental conditions, it can affect the speed at which the equilibrium coverage is attained. Thus, when the adsorption process is slow in comparison with the formation of OH_ads_, the hydrogen density must be the limiting factor. Therefore, H must be transported from a larger area toward the O-front to propagate the reaction further. A quantitative analysis can be made in terms of the effective rate for adsorption (ERA). The effective rate for adsorption, that is, how fast an active site can be refilled with H after desorption has taken place, is given, in our case, directly by *k*_1_. The ERA is in the range of 10^–15^ m^2^/s in the case of bare Ru(0001) and 10^–23^ m^2^/s for the confined reaction. When these values are compared with those of *k*_2_ for the different scenarios (10^–16^ m^2^/s), the limitation of the reaction rate under confinement by hydrogen adsorption becomes clear.

The phase diagram shown in Figure S12 of the Supporting Information (section S14), constructed from the temperature- and pressure-dependent kinetic constants describing hydrogen adsorption (*k*_1_), hydroxyl formation (*k*_2_), and water desorption (*k*_4_), allows us not only to predict the effect of confinement at variable (*p*, *T*) conditions but also to explain the differences—and apparent discrepancy—between our work and that of Wang et al.^42^ conducted at much higher hydrogen pressures. Our models indicate that, at least for the SiO_2_/Ru(001) system, confinement can act in two of the three ways described by Csicsery;^8^ that is, confinement effects can prevent reactants of getting access to the reaction site and also prevent products from leaving the reaction site. It is the relative importance of these two effects that will determine the overall kinetics of the WFR under confinement in different experimental conditions. Therefore, the scarce availability of H_ads_ at low H_2_ pressures (our work) is important for propagating the reaction cascade, whereas at high H_2_ pressures H_2_O entrapment is responsible for blocking the active sites for H adsorption and diffusion (Wang’s work^42^).

Finally, an important point derived from our study is the role of the bilayer position relative to the catalytically active ruthenium surface. Our simulations predict that the relative position can affect the reaction step responsible for the last H-addition in the cascade of steps, that is, H_2_O formation. Because none of the techniques used in our experiments are sensitive to the position of the silica film, it is virtually impossible to decide which of the scenarios investigated is experimentally more likely. For instance, from the experimental point of view, it is reasonable to assume that far in the 3O area the silica BL has its atomic position in good registry with the 3O/Ru(0001) support. On the other hand, far behind the reaction front the silica has its position in registry with the 1O/Ru(0001) surface. Thus, it is rather likely to find an intermediate region where the BL relative position changes/relaxes. Because of the rather low concentration of H_ads_ (10^–4^ ML; one H adatom in an area of 100 × 100 unit cells), we rule out hydrogen adsorption as the main driving force for the silica displacement, but its real physical origin remains unclear. This opens new opportunities for real-time studies of the dynamic processes occurring on the silica film as the reaction front propagates under the silica film. This system presents now a great opportunity for the application of the state-of-the-art high-speed scanning tunneling microscope developed in our institute.^57^

## Conclusion

We showed that the water formation reaction proceeds as a reaction front both on bare Ru(0001) and in confinement under a crystalline silica bilayer, with slower front velocities under confinement. The Arrhenius analysis of the front velocity in both confined and nonconfined environment reveals that the apparent activation energy is halved when the reaction is confined under a silica film. DFT and microkinetic simulations reproduce our experimental findings, indicating that the changes observed in the experimental values of the *E*_a_^app^ cannot be tracked down to the known transition state effects of confinement, since the transition state of the rate-determining step remains unaffected by the presence of the silica film. Interestingly, the rate-determining step of the reaction remains unchanged, being the formation of OH_ads_ after the dissociation of H_2_. On the other hand, fitting of the proposed kinetic model through a set of differential equations yields an excellent agreement with the experimental values for front velocities. Simulation of the reaction front at different temperatures allowed us to identify the H adsorption step rather than water desorption as a key parameter to reproduce the experimental apparent activation energies. The surface concentration profiles resulting from the microkinetic model indicate that while H_2_ molecules can dissociate almost anywhere on the O-poor area, water molecules (and OH as an intermediary specie) are produced only in the vicinity of the front. In this sense, it becomes clear from these results that the front width is mainly given by the concentration gradient of O_ads_ rather than H_2_O_ads_ and OH_ads_, provided that the surface concentration of the last two species is comparatively low.

## Experimental Section

The experiments were performed in the SMART microscope operating at the UE49-PGM beamline of the synchrotron light source BESSY II of the Helmholtz Centre Berlin (HZB). The aberration corrected and energy filtered instrument combines microscopy (LEEM/XPEEM), diffraction (μ-LEED), and spectroscopy (μ-XPS) techniques for a comprehensive characterization.^58−60^ The Ru(0001) single crystal was prepared by cycles of Ar^+^ sputtering and annealing in oxygen at 1170 K, until no contamination could be detected by XPS and the surface presented few 100 nm wide terraces with a sharp (1 × 1) LEED pattern. The crystalline SiO_2_ BL was used in the WFR experiments; a detailed description of the preparation procedure can be found elsewhere.^61^ For the reaction experiments, H_2_ was dosed after stabilizing the sample temperature at 540 K in UHV. Temperature-dependent measurements could be conducted once the reaction front was observed. More details can be found in the Supporting Information.

## Computational Methods

For the DFT simulations we employed the PBE-D2 functional.^62,63^ The calculations were performed by using plane wave codes Quantum Espresso^64^ and VASP^65^ with the energy cutoff of 400 eV and a 6 × 6 × 1 k-mesh. Transition states were located by using the nudged elastic band (NEB) method.^66^ In the optimizations, the position of the silica bilayer was either allowed to relax—the “optimized” case—or the *x*- and *y*-coordinates of its atoms were kept fixed in the optimal SiO_2_/3O/Ru(0001) positions—the “constrained” case. Further details can be found in the Supporting Information.

## Numerical Simulations

We propose a kinetic model in terms of a system of reaction–diffusion equations governing macroscopically the spatiotemporal evolution of the reaction fronts on the Ru(0001) surface. To compute the frontal velocity and width, the system was studied by means of dynamical system analysis and numerical simulations. The interested reader finds details on the kinetic model in the Supporting Information.

## Supporting Information Available

Experimental details; computational methods; theoretical description of kinetic constants; numerical simulations of reaction diffusion equations; LEEM snapshots of reaction front under reaction conditions; transition state structures; references (PDF)
The video S1.MP4 shows the propagation of the reaction front at 525 K. Field of view: 20 μm (MP4)
The video S2.MP4 shows the propagation of the reaction front at 550 K. Field of view: 20 μm (MP4)
The ZIP folder contains the files of transition state and intermediate structures (ZIP)
