# Supplementary material for: Insights into Reaction Kinetics in Confined Space: Real Time Observation of Water Formation under a Silica Cover
Source: J Am Chem Soc. 2021 Jun 7;143(23):8780–90. doi: 10.1021/jacs.1c03197 (PMC8297729; doi:10.1021/jacs.1c03197)
Supplement: Supplementary file 1 — ja1c03197_si_001.pdf [file ja1c03197_si_001.pdf]

Supporting Information

Insights into Reaction Kinetics in Confined Space: Real Time Observation of Water Formation under a Silica cover

Mauricio J. Prieto[1*], Thomas Mullan[2], Mark Schlutow[3*], Daniel M. Gottlob[1], Liviu C. Tănase[1], Dietrich Menzel[4,1], Joachim Sauer[2], Denis Usvyat[2*], Thomas Schmidt[1], Hans-Joachim Freund[1]

[1]*Fritz-Haber Institute of the Max-Planck Society. Faradayweg 4-6. 14195-Berlin. Germany.*

[2]*Institut für Chemie. Humboldt-Universität zu Berlin. Unter den Linden 6. 10099-Berlin. Germany.*

[3]*Institut für Mathematik. Freie Universität Berlin. Arnimallee 6. 14195-Berlin. Germany.*

[4]*Physik-Department E20. Technical University München. 85748-Garching. Germany.*

*prieto@fhi-berlin.mpg.de

*mark.schlutow@fu-berlin.de

*denis.usvyat@hu-berlin.de

Table of contents

S1. Experimental methods 3

S2. Computational methods 3

S3. Surface and bilayer geometries 4

S4. Adsorption of hydrogen onto a ruthenium surface 5

S5. Formation of water 8

S6. Desorption of water 11

S7. Diffusion on the surface 12

S8. Thermodynamic properties 13

S9. Rate constants 15

S10. Numerical simulations 17

S11. Traveling front solution 18

S12. Numerical solution of the reaction-diffusion equations 21

S13. LEEM and LEED observation of reaction waves 22

S14. Transition state structures and kinetic constants 23

References 25

S1. Experimental Methods

The experiments were carried out in the SMART microscope operating at the UE49-PGM beam line of the synchrotron light source BESSY II of the Helmholtz Centre Berlin (HZB). The aberration corrected and energy filtered LEEM/PEEM instrument combines microscopy (LEEM/XPEEM), diffraction (µ-LEED), and spectroscopy (µ-XPS) techniques for comprehensive characterization. The base pressure of the system is 10^{-10} mbar, but operation is possible at pressures up to 10^{-5} mbar of reactive gases in a temperature range between 150 and 1500 K.[1-3]

The Ru(0001) single crystal was prepared by cycles of Ar^+ sputtering at room temperature and annealing in oxygen at 1170 K. Cleaning cycles were repeated until no contamination could be detected by XPS, with terraces a few 100 nm wide and a sharp (1×1) LEED pattern. Sample temperature was measured either by a W26%Re/W5%Re thermocouple or by a pyrometer (IMPAC IGA 740) with an absolute accuracy of ±10 K. Oxygen (99.999%) and hydrogen (99.999%) were dosed directly into the experimental chamber; Si was sublimated from a 4 mm thick rod (99.999%) using a commercial evaporator (Omicron EFM3) under grazing incidence of 20°.

For all experiments a crystalline SiO_2 BL was produced and a detailed description of the preparation procedure can be found elsewhere.[4] However, for the sake of clarity it is important to mention that after the last oxidation and structure forming step O_2 intercalates into the space confined between the silica bilayer and the Ru(0001) support, thus forming a well-ordered 3O adlayer on Ru with an estimated coverage of 0.75 ML. The term 3O refers to the number of oxygen adatoms present in a (2×2) cell, as described previously in the thorough investigation of the O-phases existing on the bare Ru(0001) surface.[5-6] This state is known as the O-rich phase and corresponds to the initial stage of our water formation reaction experiments, both in the confined or the non-confined case. For the H_2 intercalation and oxidation experiments, the as prepared SiO_2/Ru(0001) sample was heated up to 540 K in UHV and only after temperature stabilization H_2 was dosed into the experimental chamber up to 1×10^{-6} mbar. Once the reaction front was observed, the temperature dependent measurements were carried out by adjusting the sample temperature by thermal radiation of a filament from the backside of the sample.

S2. Computational Methods

To investigate the relevant parts of the potential energy surface (PES), we employed Kohn Sham density functional theory[7] within the generalized gradient approximation in form of the Perdew-Burke-Ernzerhof (PBE) functional[8] with an empirical atom-pair-based dispersion correction D2 by Grimme[9]. Electronic structure calculations were performed using plane wave codes VASP Grimme[9]. Electronic structure calculations were performed using plane wave codes VASP [10] and Quantum Espresso.[11] The frozen core was represented by scalar-relativistic ultra-soft pseudo-potentials (Quantum Espresso) or the projector augmented wave method (VASP). Integration of the first Brillouin zone was done using a weighted uniform k-point grid (6×6×1 for a (2×2) cell) as proposed by Monkhorst and Pack.[12] The standard plane-wave energy cutoff of EPW = 400 eV

was used throughout the calculations. To improve the convergence in the zero-gap system, unoccupied states were partially populated by Gaussian smearing with a width of 0.15 eV. The minima on the PES were optimized until all components of all forces were less than 5×10^{-3} eVÅ^{-1} and the change in electronic energy between two optimization steps was less than 1×10^{-4} eV. Vibrational modes were calculated within the harmonic approximation using central finite-differences. Transition states were located using the nudged elastic band (NEB) method.[13] The convergence tolerances on the projected forces in the NEB optimizations were 0.05 eVÅ^{-1}. The saddle points were refined by the dimer-method.[14] In the optimization of the structures two different approaches were used. In the so-called "optimized" silica bilayer, the position of the silica bilayer was allowed to relax during the optimization process. On the other hand, in the so-called "constrained" silica bilayer, its position was kept fixed across all reaction steps, with the initial position corresponding to that having the lowest energy for the SiO_2/3O/Ru(0001) system.

S3. Surface and bilayer geometries

The topic of this study is the catalytic property of a ruthenium (0001) surface with respect to the water-formation reaction. Our corresponding computational model was obtained from an optimized hexagonal close-packed ruthenium bulk structure, from which a repeated 3-layer slab with a (0001) surface direction and a vacuum layer of 30 Å was constructed. The optimized and experimental bulk lattice constants are given in Table S1. If not stated otherwise the (2×2) surface supercell was used in the calculations. The top ruthenium layer was allowed to relax while the bottom two were fixed at their bulk positions. A representation of the surface slab with and without a bilayer on top is shown in Figure S1.

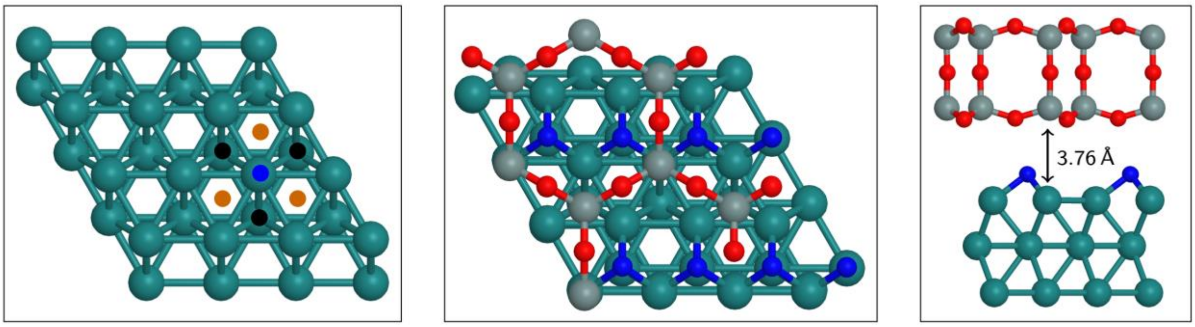

Figure S1. Left: On-top view of the bare ruthenium (0001) surface. Colored dots indicate binding sites: "on-top" (blue), "hcp" (orange) and "fcc" (black). Middle and right: Top and side view of a ruthenium surface with a 2O oxygen coverage and a SiO_2 bilayer within the constrained optimization. Surface oxygens are shown in blue for distinction from the bilayer. A (4×4) supercell is shown here.

In the following we analyze the energetics of the water formation reaction on the ruthenium surface by means of minimal energy pathways (MEPs) for the complete reaction (discussed in the main text), as well as the MEPs for the individual steps (analyzed below). These steps include adsorption of hydrogen, formation of OH groups, formation of water molecules and desorption of the water molecules from the Ru-surface. The MEPs were calculated for 1O and 2O oxygen coverages with and without presence of the silica bilayer. In order to discuss the situations where the bilayer position with respect to the surface configuration is favorable or unfavorable at each reaction step, we considered (i) a fully optimized bilayer (FOB), and (ii) a bilayer only optimized in the z coordinate (ZOB), while the x and y coordinates were frozen, hereafter referred to as optimized and constrained bilayer, respectively. Below we provide a more detailed description. To be able to compare the energetics between all different cases and regimes, in the plots of the complete MEP as well as the MEPs for the individual steps, the energy of the leftmost reactant state is always taken as the zero-reference energy.

Table S1. Experimental[15] and calculated ruthenium bulk unit cell parameters

	$\|\vec{a}\|/a_0$	$\|\vec{c}\|/\|\vec{a}\|$
Theoretical	5.1337	1.56
Experimental	5.1132	1.58

S4. Adsorption of hydrogen onto a ruthenium surface

Experimentally, the ruthenium surface is already partially covered with atomically bound oxygen, before it is exposed to hydrogen gas, where both species are known to form ordered adlayers on the surface at low enough temperatures.[16] As reported in the literature, oxygen prefers hcp-hollow binding-positions, while hydrogen will bind to fcc-hollow sites.[17-18] However, when oxygen atoms are present, the actual position of hydrogen is determined by a balance between the attraction to the surface and strong repulsion at a short distance between oxygen and hydrogen. As a consequence, on-top binding sites also become available for hydrogen when it is symmetrically surrounded by repelling oxygen atoms and therefore maximizes the distance to these atoms. Since oxygen binds to hcp-hollow sites, all adjacent fcc-hollow sites become unavailable, forcing hydrogen to either bind to hcp-hollow or on-top sites (Figure S2). In general, the adsorption process of hydrogen onto a ruthenium surface is determined by two subsequent steps: First, the incoming molecule approaches the surface, before it dissociates into two hydrogen atoms. The dissociation happens stepwise (e.g. one hydrogen atom remains on the on-top site while the first is moving to the hcp adsorption site). Since the surface oxygen tends to repel the adsorbed hydrogen atoms, an increase of oxygen coverage impedes adsorption of hydrogen, as the necessary on-top sites for splitting are getting too close to oxygen atoms.

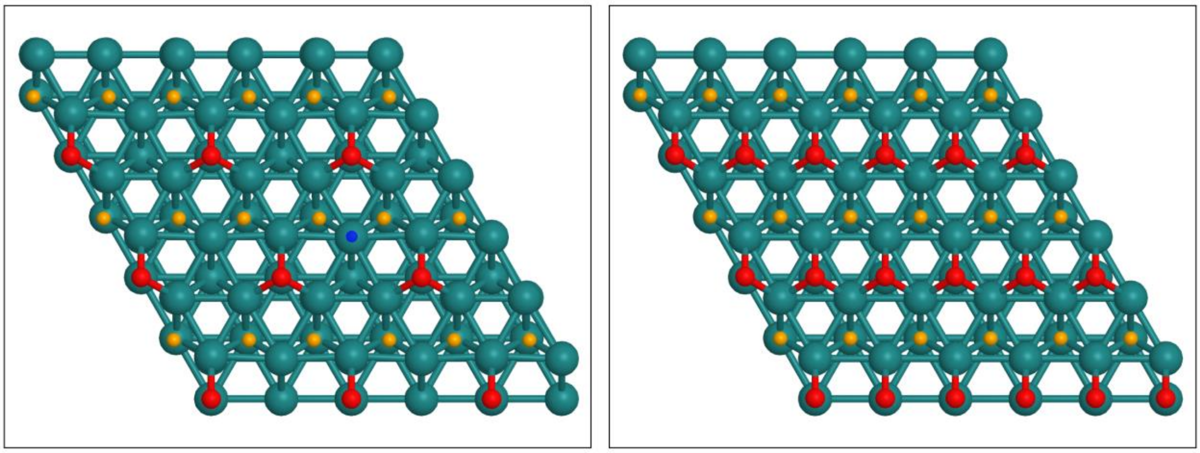

Figure S2. (2×2)-1O (left) and (2×2)-2O (right) surfaces with two hydrogens (orange) adsorbed per unit cell. Hydrogen is forced to occupy hcp hollow sites to maximize its distance to adjacent oxygen (red) atoms. For the (2×2)-1O surface, there is one additional on-top binding site available surrounded by 3 oxygen atoms (indicated by a blue dot).

As can be seen from the calculated MEP (Figure S3), the electronic part of the hydrogen adsorption onto a bare surface with low oxygen concentration is barrierless and the gain in potential energy due to adsorption outweighs the cost of splitting the hydrogen molecule. When increasing the oxygen coverage on the surface, some of the on-top sites required for splitting H_2 are in close vicinity to an oxygen atom, resulting in a slightly activated process. For coverages higher than 0.75 ML (3 oxygens per (2×2) unit cell), dissociative adsorption becomes essentially impossible since there are not enough free binding sites neighboring each other.

The addition of a van-der-Waals-bound SiO_2 bilayer over the surface converts these adsorption processes into clearly activated processes, irrespective of the oxygen concentration on the surface. The penetration through the lower silica ring becomes the decisive energy barrier. It is strongly influenced by the nearby oxygens on the surface, as well as by the position of the bilayer, relative to the on-top site required for splitting.

It is difficult to predict the position of the SiO_2 rings with respect to the surface and oxygen atoms in real experiments because irregularities in the bilayer and/or surface may lead to a shift of these rings relative their optimal position on an ideal surface. In order to cope with this problem, as noted above, we modeled two distinct (extreme) cases: an optimized and a constrained bilayer. In the constrained-bilayer case, the x- and y-coordinates of the bilayer were chosen to coincide with those of its optimal position on the Ru surface with a 3O coverage (0.75 ML), corresponding to the in situ preparation of the bilayer.[19] With this position of the bilayer fixed, the coverage was reduced to 2O – to model the reaction (this coverage is expected in the active reaction region), and to 1O – to model the hydrogen adsorption. In the optimized-bilayer case, starting from the initial 3O geometry the bilayer was allowed to find its optimal position on the surface by full relaxation at each step along the adsorption and reaction paths.

The adsorption processes for different cases are shown in Figure S3. One can note a higher barrier and a less negative adsorption energy in the optimized-bilayer 1O case compared to the constrained one (note that although the absolute energies in optimized-bilayer calculations are obviously lower than those of the constrained ones, this does not have to hold for the relative energies). Here it is attributed to an additional movement of the bilayer, needed for the incoming H_2 molecule to reach an on-top site. In the constrained case, the bilayer is from the onset positioned in such a way that the on-top site for H_2 is readily available. This effect is virtually absent in the 2O case, as the positions of the constrained and optimized bilayer are similar, and all on-top sites are inconveniently located not far from an oxygen atom.

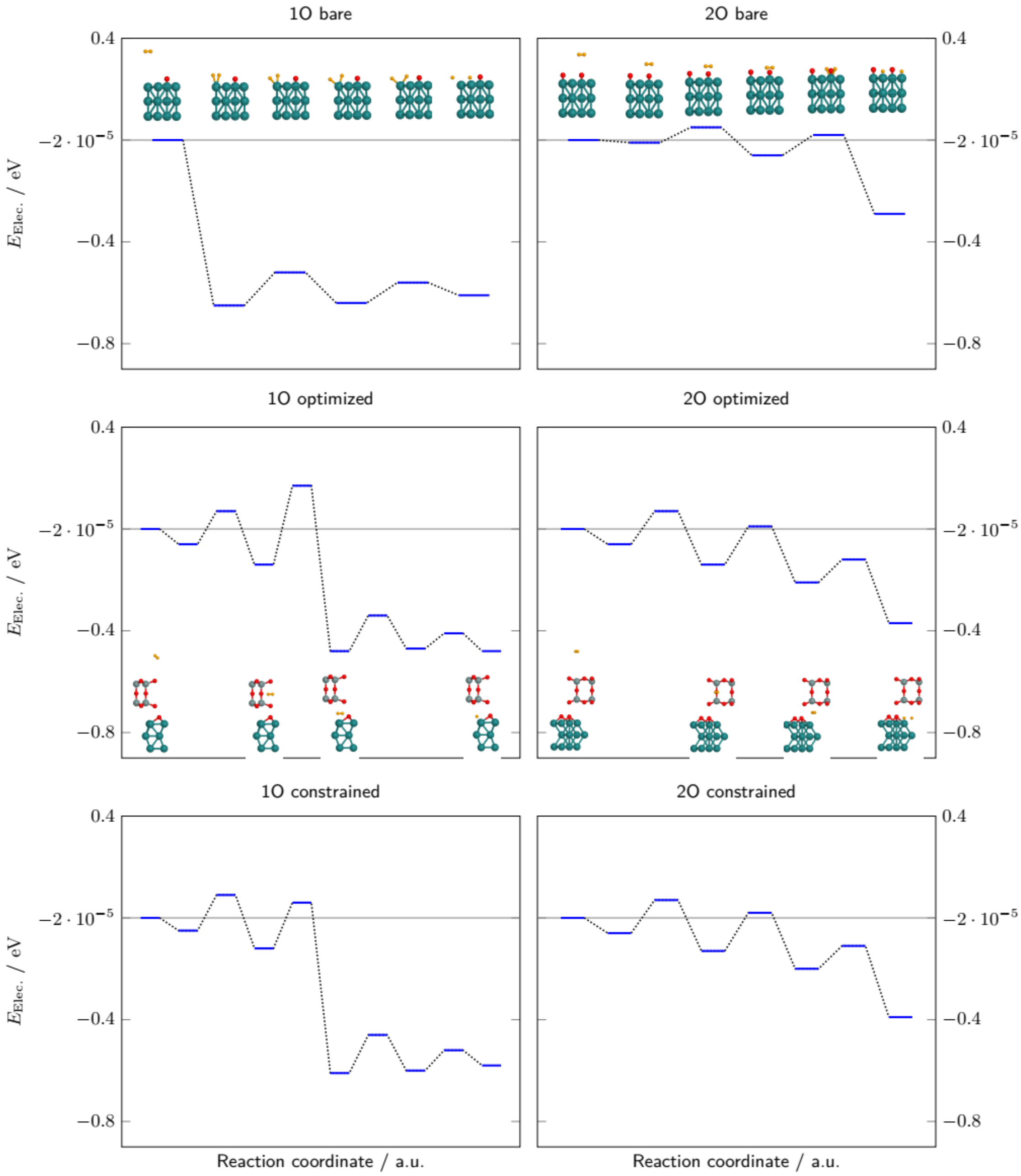

Figure S3. Comparison of the different MEPs for the adsorption of molecular hydrogen onto a ruthenium surface with different oxygen coverages.

S5. Formation of water

In order to model the formation of water, we consider three elemental reaction channels on the surface:

$$O* + H* \rightleftarrows OH* \tag{S1a}$$

$$OH* + H* \rightleftarrows H2O* \tag{S1b}$$

$$2\ OH* \rightleftarrows H_2O* + O* \tag{S1c}$$

The disproportionation reaction (S1c) is in competition with the water formation reaction (1b), but only takes effect when there is a considerable amount of OH present on the surface. Also, the reverse reaction of eq. (S1c) – the comproportionation – competes with reaction (S1a) in producing OH, but again it can provide a considerable contribution only if the concentration of water on the surface is sufficiently high.

Figure S4 shows the MEPs for the six different systems of interest for the first two reactions discussed above. The first step, corresponding to the formation of OH from atomically adsorbed O and H, features a large electronic barrier that is mostly independent of the chemical environment. This can be explained by the relatively large size of the SiO_2-bilayer rings, enclosing multiple reaction sites in such a way that there will be at least one mostly undisturbed site available for the reaction. Additionally, neither surface hydrogen nor surface oxygen show considerable interaction with the bilayer due to their small size and absence of hydrogen-bridge bonding interactions.

In contrast to this first reaction step, the formation of water from OH and H is energetically much more favorable on a bare 2O surface or one equipped with a free moving silica bilayer. The uniformly lower activation energy and increased stability of water species on a 2O over a 1O surface suggests that, the higher the oxygen coverage on the surface, the weaker it is bound to it. The hydrogen bonding between the water molecule and the remaining surface oxygen also contributes to the energy gain in the 2O case. We also note that in the constrained-bilayer structure the barrier and the energy for the water molecule formation is noticeably higher than in the optimized-bilayer or bare cases, especially for the 2O initial coverage. Formation of water molecules inside these rings limits the available space below the bilayer, which becomes most prominent in the 2O case, with one oxygen atom remaining on the surface. Therefore, without the horizontal optimization of the bilayer, which can open favorable on-top position, the energy for both the transition state and the formed water molecule remains high by comparison.

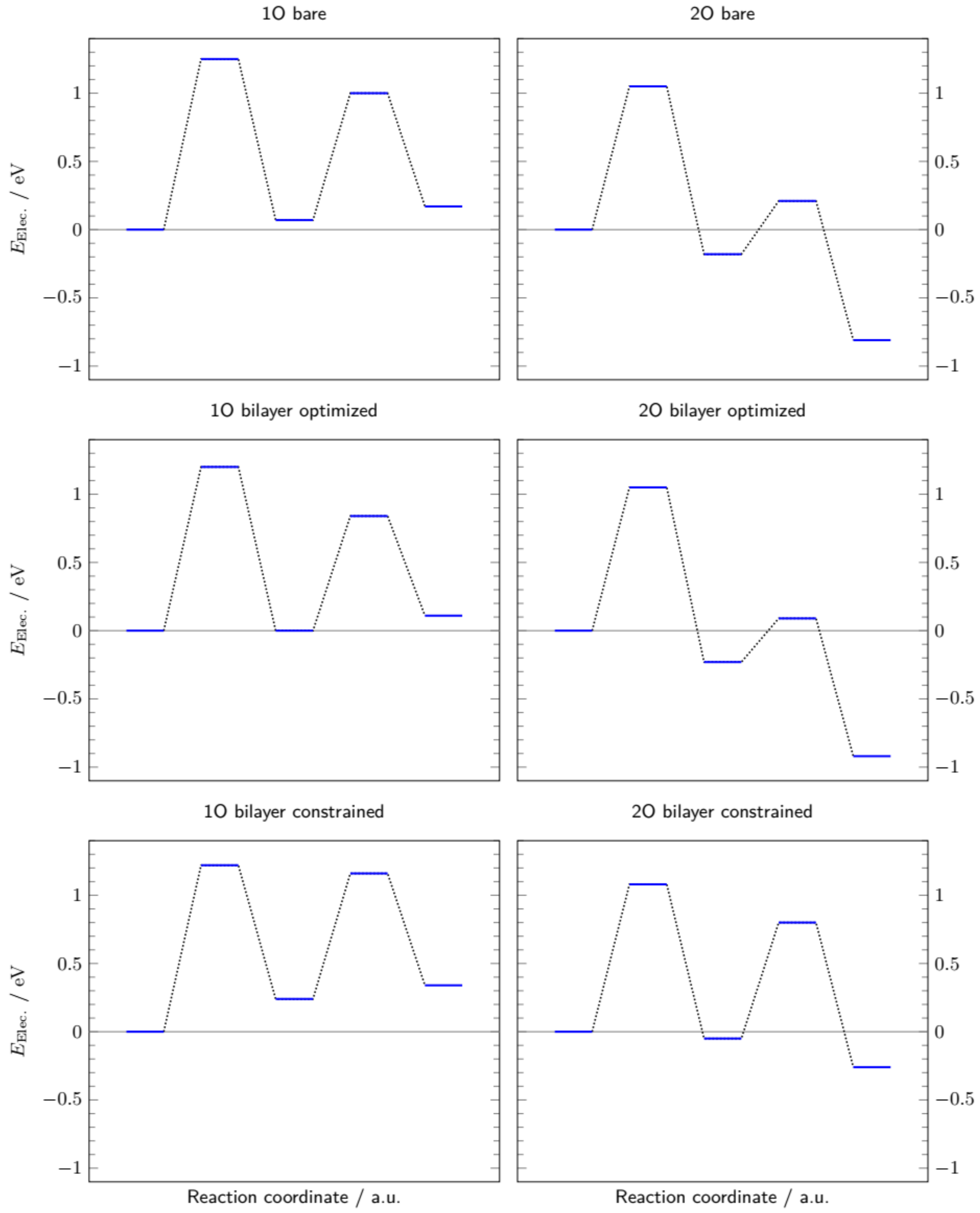

Figure S4. Comparison of the different electronic minimum energy pathways for the reaction cascade 2H∗ + O∗ ⇄ H∗ + OH∗ ⇄ H_2O∗. The first step is virtually independent of its chemical surroundings while the formation of water becomes strongly dependent on the presence of free on-top binding sites.

The electronic MEP for the formation of water from two OH groups eq. (eq. (S1c), disproportionation) is shown in Figure S5 on the left. We split the reaction into two steps, a "diffusion" step and a "reaction" step, since the lowest energy configuration of two OH groups is separated by one higher minimum on the PES. It can be seen that the electronic barriers are small in comparison to the formation of OH groups and consist mainly of bringing two hydroxyl groups close together. Consequently, this reaction channel can indeed increase the rate of water formation but only if there is a non-negligible probability of two OH groups appearing close to each other. However, based on the estimated OH surface concentrations (see main text) and the rather large energy diffusion barrier for this species (in comparison with that of H) we disregard this channel.

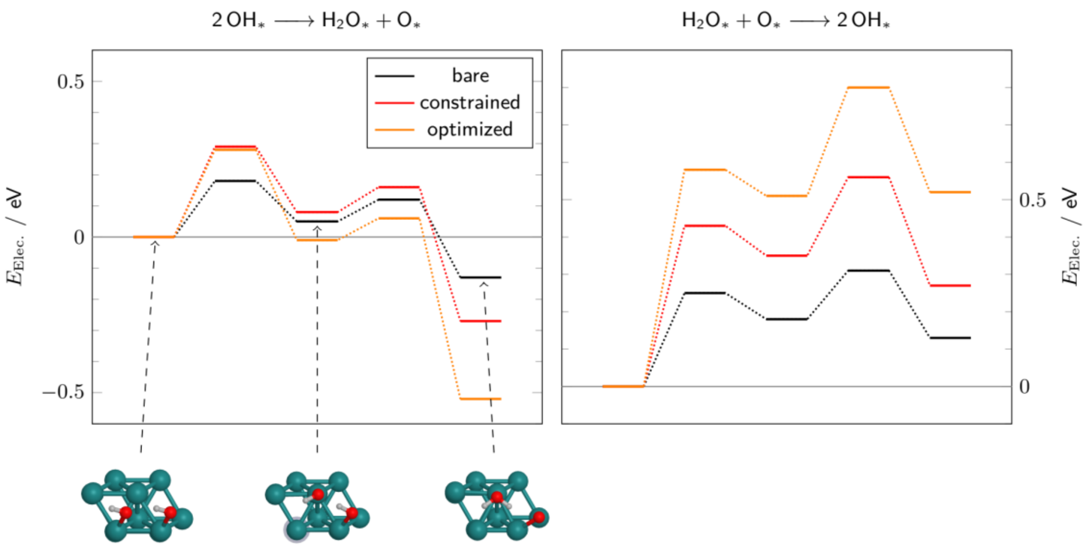

Figure S5. Comparison of the different electronic minimum energy pathways for the reaction 2OH∗ ⇄ H_2O∗ + O∗. The left diagram shows the disproportionation reaction, the right shows the comproportionation reaction, i.e., the reverse reaction. For both plots the energies are referred to the initial state.

The comproportionation reaction of eq. (S1c) allows the system to bypass the rate-limiting step (S1a) and form two OH groups at one instance. Again, our calculations show that, even for the confined case, the concentration of water on the surface is low (3.9×10^{-15} ML on bare ruthenium at 500 K), hence this process is most likely not dominant. Besides, the barrier for the comproportionation reaction (Figure S5 right) in the presence of the bilayer is of a considerable magnitude (for the optimized-bilayer case it is as much as 0.8 eV), so for the adsorbed water we do not expect this reaction to occur at high frequency. Furthermore, the barrier for the disproportionation reaction is rather low, while at the same time the OH diffusion is expected to be slow (vide infra). Consequently, the chance of two OH groups, which were created by the dispropotionation channel in close vicinity, reacting back to form water is high, giving hydrogen atoms little chance for attacking one of them.

S6. Desorption of water

At higher concentration, H_2O molecules can form intricate hydrogen-bonding networks resulting in an increase in desorption energy.[20] However, at low concentrations, these networks are not possible and the average residence time of water on the surface is expected to decrease. Figure S6 shows the MEPs for the water desorption for the six different cases at consideration. The desorption energies for a single water molecule from a bare surface with a concentration of 1O and 2O per (2×2) unit cell are 0.67 eV and 0.91 eV, respectively. The increase in the desorption energy can be explained by the additional H-bonding that is possible with one O remaining on a 2O surface. At high temperatures however, due to the entropic effects, the desorption energy becomes negative (see main manuscript) and water molecules are expected to leave the bare surface virtually instantaneously. Similarly to the hydrogen adsorption, the addition of a 2D confinement introduces two additional transition states, one for passing each SiO_2 layer. Here the highest transition state corresponds to the upper ring of the bilayer. It also should be noted that a fixed bilayer already includes the increase in potential energy of the water molecule bound to the surface as discussed above, which effectively lowers the barrier of desorption in comparison to a free moving bilayer or unconfined surface.

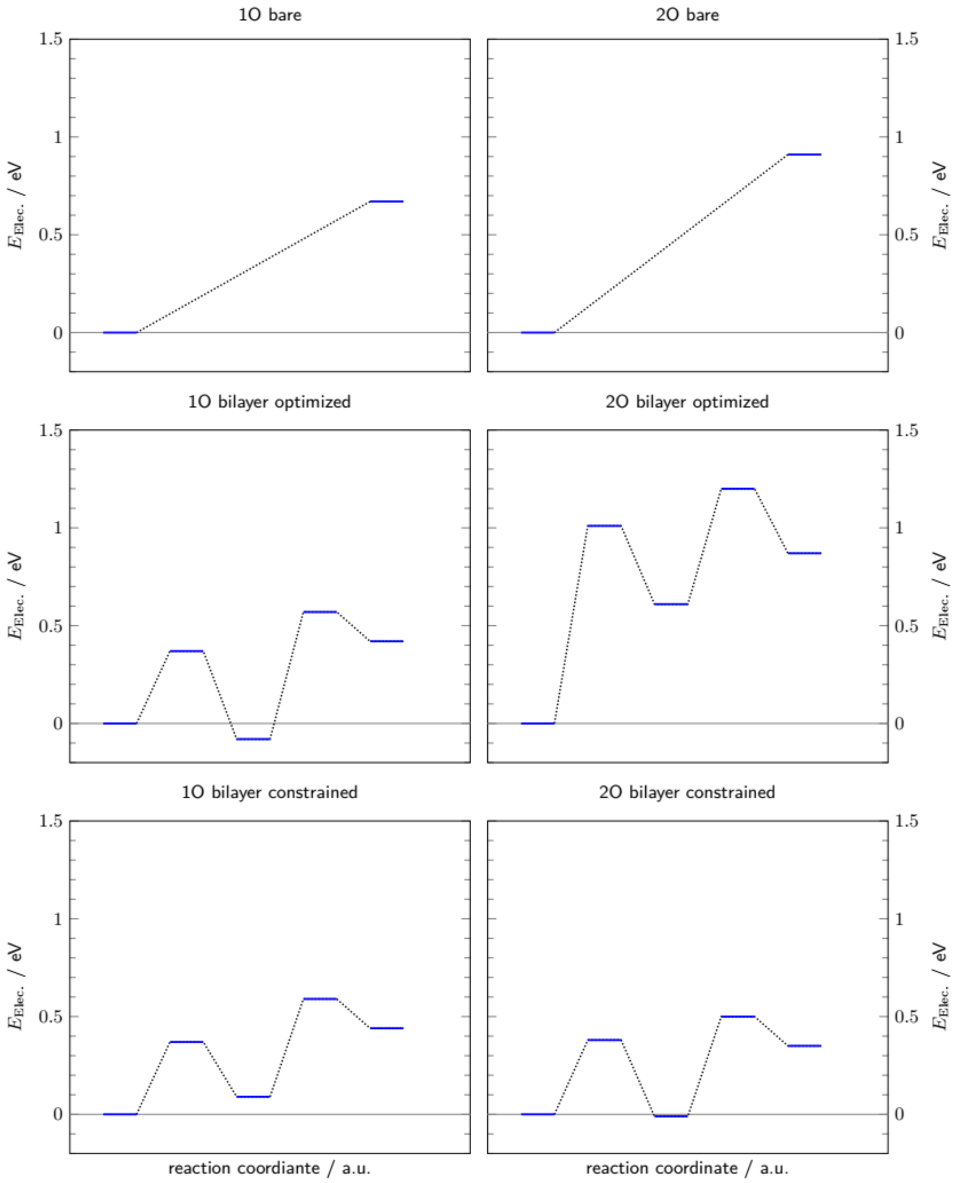

Figure S6: Comparison of the different electronic minimum energy pathways for desorption of H_2O from a Ru(0001) surface with various concentrations of oxygen.

S7. Diffusion on the surface

In this section we consider diffusion of hydrogen atoms and hydroxyl (OH) groups on the ruthenium surface, since the diffusion rates of these species can strongly influence the overall reaction mechanism. We start with the diffusion of atomic hydrogen. Unfortunately, there is a vast number of possible different diffusion channels. In order to elucidate possible diffusion barriers, we consider a single possible route on the ruthenium surface with a 1O oxygen coverage within a (2×4) cell. A representation of the diffusion path and the corresponding electronic energy diagram is shown in Figure S7. It is apparent that with barriers between 0.1 and 0.2 eV, hydrogen is able to diffuse for sufficiently long distances at our experimental temperatures. OH diffusion, on the other hand, exhibits slightly increased barriers of around 0.4 eV. However, this

barrier grows notably when OH tries to enter a 2O region (Figure S7 middle and right). Neither OH, nor Hydrogen diffusion showed a strong dependence on the presence of the bilayer.

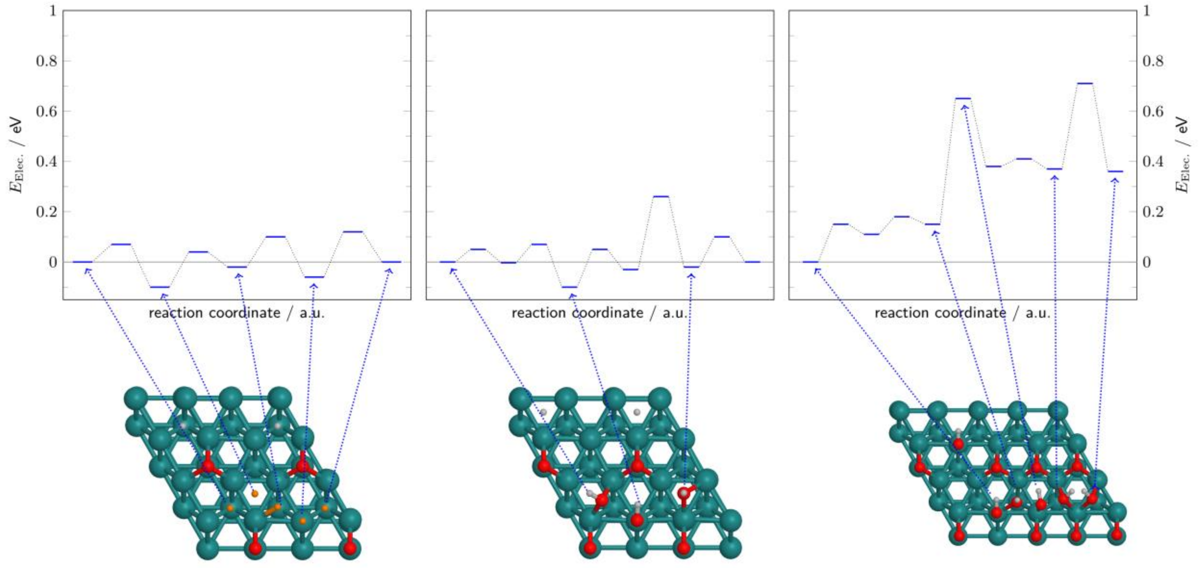

Figure S7. Middle and left: MEPs for the diffusion of atomic Hydrogen (left) and OH groups (right) on a bare 1O Ruthenium surface. The path completes one (2 × 2) unit cell and wraps around itself. Right: MEP for the diffusion of OH groups across a 1O–2O domain boundary

S8. Thermodynamic properties

We only give a short outline of the thermodynamic formulation required for the description of our system. Further information can be found in the relevant literature.[21]

Our goal is to compute the standard Gibbs free energy G^0 (we drop the superscript 0 for simplicity) for all points on the potential energy surface (PES) that are relevant for the reaction kinetics. We start from the standard definition of the Gibbs free energy

$$G\,(T,p) = H - TS \tag{S2a}$$

$$= U + pV - TS \tag{S2b}$$

with the enthalpy H, internal energy U, entropy S, Volume V (all per particle), pressure p and temperature T, which is minimized in any system under isothermal ($\mathrm{d}T = 0$) and isobaric conditions ($\mathrm{d}p = 0$). For any reaction only including condensed matter, the contribution pV will be negligible, since $V_{gas} >> V_{condensed} \approx 0$, while for steps including particles in the gas phase we invoke the ideal gas law and set $pV = k_B T$, where k_B is Boltzmann's constant. Internal energy U and entropy S are readily available from the total partition function q_{tot} of the system and its internal energy at zero temperature $U_{T=0\,K} = E_{electronic} + \frac{1}{2}\sum_i \varepsilon_i$, where ε_i is the energy of the i-th vibrational mode:

$$U = k_B T^2 \frac{\partial}{\partial T} \ln q_{tot} + U_{T=0K} \qquad \text{(S3)}$$

$$S = k_B \ln q_{tot} + k_B T \frac{\partial}{\partial T} \ln q_{tot} \qquad \text{(S4)}$$

Plugging eqs. (S3) and (S4) into eq. (S2b), we arrive at:

$$G = \begin{cases} U_{T=0K} - k_B T \ln q_{tot} + k_B T & (gas\ phase) \quad \text{(S5a)} \\ U_{T=0K} - k_B T \ln q_{tot} & (condensed\ phase) \quad \text{(S5b)} \end{cases}$$

The real total partition function q_{tot}, which is required to calculate the enthalpy and entropy according to eqs. (S3) and (S4) is approximated as a product of the individual contributions from vibration, translation and rotation of the system under study:

$$q_{tot} = \prod_i q_i = \begin{cases} q_{trans}^{3D} \cdot q_{rot} \cdot q_{vib} & (gas\ phase) \quad \text{(S6a)} \\ q_{vib} & (condensed\ phase) \quad \text{(S6b)} \end{cases}$$

where *i* runs over all degrees of freedom. Again, for any system only including condensed matter, there are no more free translations or rotations possible, while for the gas phase we approximate the rotational and translational contribution by using the standard partition functions of a symmetric, rigid free gas particle of mass *m*:

$$q_{trans}^{3D} = \frac{V}{\Lambda^3} = V \cdot \frac{(2\pi\, m\, k_B T)^{\frac{3}{2}}}{h^3} = \frac{(k_B T)^{\frac{5}{2}}}{p} \times \frac{(2\pi\, m)^{\frac{3}{2}}}{h^3} \qquad \text{(S7)}$$

$$q_{rot} = \begin{cases} q_{rot}^{linear} = \dfrac{8\pi^2 I k_B T}{\sigma h^2} & \text{(S8a)} \\ q_{rot}^{general} = \left(\dfrac{8\pi^2 k_B T}{h^2}\right)^{\frac{3}{2}} \cdot \dfrac{\sqrt{\pi\, I_A I_B I_C}}{\sigma} & \text{(S8b)} \end{cases}$$

where *h* is Planck's constant, I is the moment of inertia of a linear molecule, I_A, I_B and I_C are the diagonal components of the inertia tensor for any molecule and σ is a symmetry factor ($\sigma_{H2O} = \sigma_{H2} = 2$). The vibrational contribution is given by

$$q_{vib} = \prod_i \left[1 - exp\left(-\frac{\varepsilon_i}{k_B T}\right)\right]^{-1} \qquad \text{(S9)}$$

where ε_i is the energy of the i-th vibrational eigenmode. Note, that we do not include the electronic degrees of freedom in the partition function, since we assume that only the electronic ground state is populated and its contribution is included in the term $U_{T=0\,K}$ within the calculation of the free energy.

In calculations involving weakly interacting species, some of the harmonic vibrational frequencies turn out to be very close to zero, unphysically blowing up the entropic contribution, or even imaginary, leading to inconsistencies in the entropy along the reaction path. In our calculations this problem occurred for the vibrations originating from the rotations of the hydrogen molecule at the adsorption transition states, corresponding to its penetration through the bilayer. In these calculations we did not include these vibrations in the partition function (S9) but added the rotational partition function instead. Besides, to obtain balanced entropic contributions, in the optimized case, the vibrations of the bilayer in the x-and y-directions were not included in the vibrational partition function (S9) at any reaction step. Firstly, these vibrations are essentially anharmonic and of very low frequency, so within the harmonic approximation their contribution to the entropy would be substantial but very inaccurate. Meanwhile, in the constrained case, these vibrations are entirely absent, as in this case the bilayer is not allowed to move in the x- or y-direction (vide supra).

S9. Rate constants

Our microscopic kinetic model includes the following reaction steps:

$$H_2 + 2* \underset{k_{-1}}{\overset{k_1}{\rightleftarrows}} 2\,H_* \tag{S10a}$$

$$H_* + O_* \underset{k_{-2}}{\overset{k_2}{\rightleftarrows}} OH_* + * \tag{S10b}$$

$$H_* + OH_* \underset{k_{-3}}{\overset{k_3}{\rightleftarrows}} H_2O_* + * \tag{S10c}$$

$$H_2O_* \overset{k_4}{\rightarrow} H_2O \quad + * \tag{S10d}$$

and diffusion of hydrogen atoms on the Ru surface. Here k_i are the corresponding rate constants and $*$ denotes a binding site on the surface. On the basis of the arguments presented above we disregard the disproportionation and comproportionation paths (Equation (S1c)), as well as diffusion of OH and H_2O molecules on the surface, due to the high diffusion barriers found. Note, that oxygen is present on the surface before hydrogen is allowed to reach the surface and has therefore a predetermined maximum coverage at time t = 0 s.

For calculation of the rate constants, we utilize Transition State Theory and the Eyring equation

$$k = \frac{k_b T}{h}\frac{q^{\ddagger}}{q} = \frac{k_b T}{h}\, e^{-\frac{\Delta G^{\ddagger}}{k_b T}} \tag{S11}$$

where ‡ denotes the transition state.

The direct and reverse reactions (S10a–S10c) are of second order, apart from the dissociative adsorption of hydrogen (S10a), described by k_1, which is formally of third order. The rate

equation involving k_1 depends therefore not only on the concentration of free sites on the surface (squared), but also on the concentration (or pressure) of H_2 in the gas phase. Since the hydrogen pressure is constant throughout the reaction, we consider it as standard reference pressure. Then renormalization of k_1 for the rate equation (S6) in the main text requires division by standard reference concentration or pressure, which cancels this dependence, resulting in a quasi-second-order reaction. However, we note that a linear pressure dependence still remains within k_1: Due to the loss of the translational degree of freedom in the transition state, in $\frac{q^{\ddagger}}{q_{H_2}}$ the pressure factor of q_{H_2} (eq. (S6)) is no longer compensated.

For the second-order reactions, the rate equations (S6)–(S9) within the main text involve the quadratic dependence on the surface concentrations. Therefore, the rate constants k_1, k_{-1}, k_2, k_{-2}, k_3 and k_{-3} have to be normalized with respect to a standard surface concentration. For the latter we choose one particle per (2×2)-cell. The reaction (S10d) is of first order, so no renormalization of k_4 is required.

Several steps involve two or more consecutive barriers (compare Figure S3 in the main text). In particular, this is the case for adsorption and desorption of hydrogen and water through the bilayer, where an additional minimum occurs when the molecule is trapped in the middle of the cage. For the hydrogen case, the main barriers are noticeably higher than the secondary ones, so for k_1 and k_{-1} the highest energy point was considered as the transition state, which was then used to calculate the respective Gibbs free energy.

In the constrained-bilayer case, the water desorption features two transition states with similar energy and a comparably deep intermediate minimum. Therefore, we expect that once the water molecule penetrates the first layer of the silica film it will temporarily be trapped in the cage until it finally penetrates the second layer and desorbs. Hence, we neglect the re-adsorption of water molecules onto the surface and calculate k_4 using the first maximum as the TS.

Examining the water desorption through an optimized bilayer, the situation is somewhat different: the initial barrier is noticeably higher and the minimum for the trapped water is shallow in comparison, while the second barrier is again rather high. Consequently, the probability of the water molecule bouncing back onto the surface is increased in comparison to the constrained-bilayer case. In this situation, for the calculation of k_4 we employ the theory developed in ref.[22], where our double barrier process can be represented by the following formal reaction:

$$H_2O_* \underset{k_{-e}}{\overset{k_e}{\rightleftarrows}} H_2O_{cage} \overset{k_f}{\rightarrow} H_2O_{gas} \qquad \text{(S12)}$$

Then the effective k_4, that includes the possibility of readsorption, can be approximated as:

$$k_4^{eff} = \frac{k_e k_f}{k_e + k_{-e} + k_f} \qquad \text{(S13)}$$

As for the bare surface, at the experimental temperatures water desorption is a barrierless process. As a result, for this case we approximated k_4 simply by setting it to $k_4 = \frac{k_B T}{h}$.

Finally, we discuss hydrogen diffusion on the surface. The general expression for the diffusion coefficient of a particle, whose movement is hindered by a barrier of magnitude $\Delta G^{\ddagger}$, is given by

$$D = \frac{\alpha^2}{4} \frac{k_B T}{h} e^{-\frac{\Delta G^{\ddagger}}{k_B T}} \tag{S14}$$

where α is the hopping distance from one minimum to the next. In our case the diffusion of hydrogen from one cell to another features 4 non-equivalent barriers (comp. Figure S7), at least for the chosen diffusion path. However, since the barriers are of comparable height, we take $\Delta G^{\ddagger}$ to be an arithmetic mean of those. The ZPE and thermal contributions are expected to be similar along the diffusion path, hence we approximated the free-energy differences $\Delta G^{\ddagger}$ in this case by their electronic contributions only ($\Delta G^{\ddagger} \approx \Delta E^{\ddagger}$). As the hydrogen diffusion is comprised of four individual diffusion steps within a cell, the hopping distance is chosen as $\alpha = \frac{1}{4} a_0$, where a_0 is the (2×2)-cell parameter.

S10. Numerical Simulations

We propose a kinetic model in terms of a system of coupled nonlinear partial differential equations that govern the spatio-temporal evolution of the concentrations of hydrogen, oxygen, hydroxyl and water on the Ru(0001) surface. This kinetic model is introduced in the discussion section of the main text, in terms of a reaction-diffusion model.

Before solving the equations numerically, they were studied analytically in terms of dynamical system analysis to find estimates for the frontal velocity and width. It was deduced that a steady traveling solution must consist of a front in the oxygen concentration, a back in the hydrogen concentration and pulses for the hydroxyl as well as water concentration. Remarkably, standard techniques like linear stability analysis and the leading-edge approach[23] either failed or were not applicable to compute estimates for the frontal velocity. Hence, the numerical solutions were all the more valuable. To solve the equations numerically we applied the robust and efficient methods described in detail in the next sections.

The numerical scheme is based on the operator splitting method that allows for solving the reaction and the diffusion part successively.[24] Both the diffusion and reaction part are solved by implicit methods which may be computationally more expensive but ensure stability as the system involves various, highly different time scales rendering the equations extremely stiff.[25] We chose Neumann boundary conditions and an a-priori estimated solution as initial condition similar to a nucleus that ignites the frontal formation. The equations were spatially discretized on a grid with 800 grid points and temporally up to 10^9 steps depending on the convergence behavior. On a computational cluster equipped with nodes of Intel X5650 cores, each computation terminated in a few minutes up to several hours depending on the set of kinetic rates from the DFT simulations.

S11. Traveling front solution

In this section, we analyze a chemical reaction front described by the system of reaction-diffusion equations introduced in equations 6-10 of the main text. Mathematically, the system represents a coupled set of nonlinear partial differential equations. It exhibits two spatially homogeneous equilibrium states or stationary solutions when ignoring time derivatives. The first equilibrium condition corresponds to the situation where H, OH and H_2O concentrations vanish while O concentration reaches its maximum. The second equilibrium corresponds to the vanishing of O, OH and H_2O concentration while H concentration reaches a maximum. We write all concentrations of species participating in the reaction as the state vector

$$\boldsymbol{n} = \begin{pmatrix} n_H \\ n_O \\ n_{OH} \\ n_{H_2O} \end{pmatrix}. \tag{S15}$$

Then, the equilibrium condition in front and behind the front is formulated as

$$\boldsymbol{n}^- = \begin{pmatrix} \alpha n^0 \\ 0 \\ 0 \\ 0 \end{pmatrix} \text{ and } \boldsymbol{n}^+ = \begin{pmatrix} 0 \\ n^0 \\ 0 \\ 0 \end{pmatrix} \tag{S16}$$

where n^0 is the maximum density of adsorption sites and the small value $\alpha = \frac{1}{1+\sqrt{\frac{k_{-1}}{k_1}}}$ is the fraction of these sites occupied by H. By applying linear stability theory, it can readily be shown that both equilibria are dynamically stable thus rendering the whole system as bistable. Due to this property, the leading-edge approach to find the marginal front velocity (see ref [23] and references therein) is not applicable. The system of reaction-diffusion equations may be written more compactly in vector form as:

$$\frac{\partial \boldsymbol{n}}{\partial t} = \boldsymbol{F}(\boldsymbol{n}) + \mathbf{D}\frac{\partial^2 \boldsymbol{n}}{\partial x^2} \tag{S17}$$

where $\mathbf{D}$ = diag(D_H, 0, 0, 0) is a diagonal matrix denoting the diffusion coefficient matrix and $\boldsymbol{F}$ is the vector-valued nonlinear reaction function. To construct propagating front solutions, we introduce the coordinate:

$$z = x - ct \tag{S18}$$

with x being the real space coordinate, t the time and c the experimentally detected front velocity. A propagating front is now a stationary (Galilean) solution in the co-moving coordinate z. So, the partial differential equations are reduced to a coupled set of ordinary differential equations as follows:

$$-c\,\frac{\partial n_H}{\partial z} = -2k_{-1}n_H^2 + 2k_1 n_*^2 - k_2 n_H n_O - k_3 n_H n_{OH} + D_H\frac{\partial^2 n_H}{\partial z^2} \tag{S19a}$$

$$-c\frac{\partial n_O}{\partial z} = -k_2 n_H n_O \tag{S19b}$$

$$-c\frac{\partial n_{OH}}{\partial z} = k_2 n_H n_O - k_3 n_H n_{OH} \tag{S19c}$$

$$-c\frac{\partial n_{H_2O}}{\partial z} = k_3 n_H n_{OH} - k_4 n_{H_2O} \tag{S19d}$$

$$n_* = n^0 - n_H - n_O - n_{OH} - n_{H_2O}. \tag{S19e}$$

This second order system of equations can be simplified to first order when introducing the derivative of n_H as a new prognostic variable,

$$\dot{n}_H = q \tag{S20a}$$

$$\dot{q} = \frac{2k_{-1}}{D} n_H^2 - \frac{2k_1}{D} n_*^2 + \frac{k_2}{D} n_H n_O + \frac{k_3}{D} n_H n_{OH} - \frac{c}{D} q \tag{S20b}$$

$$\dot{n}_O = \frac{k_2}{c} n_H n_O \tag{S20c}$$

$$\dot{n}_{OH} = -\frac{k_2}{c} n_H n_O + \frac{k_3}{c} n_H n_{OH} \tag{S20d}$$

$$\dot{n}_{H_2O} = -\frac{k_3}{c} n_H n_{OH} + \frac{k_4}{c} n_{H_2O} \tag{S20e}$$

$$n_* = n^0 - n_H - n_O - n_{OH} - n_{H_2O}. \tag{S20f}$$

Here, we replaced the derivative with respect to z by a dot. In what follows we consider the system of ordinary differential equations as a dynamical system. Hence, a solution representing a traveling front can be treated as a heteroclinic trajectory in the phase space spanned by the extended state vector $\boldsymbol{p} = \left(n_H, q, n_O, n_{OH}, n_{H_2O}\right)^T$ connecting the two equilibrium points denoted by

$$\boldsymbol{p}^- = \begin{pmatrix} \alpha n^0 \\ 0 \\ 0 \\ 0 \\ 0 \end{pmatrix} \text{ and } \boldsymbol{p}^+ = \begin{pmatrix} 0 \\ n^0 \\ 0 \\ 0 \\ 0 \end{pmatrix}. \tag{S21}$$

Let us define, without loss of generality due to the symmetry of the dynamics, that $\boldsymbol{p}^-$ denotes the equilibrium point at $-\infty$. Then, $c > 0$ must hold to get a meaningful solution as otherwise n_O might become negative by close inspection of eq. S20c which would render an unphysical solution. We can conclude that the solution must consist of a front in n_H, a back in n_O, and pulses for n_{OH} as well as n_{H_2O}.

Let us write the dynamical system in vector form,

$$\dot{\boldsymbol{p}} = \boldsymbol{G}(\boldsymbol{p}) \text{ where } \boldsymbol{G}(\boldsymbol{p}^-) = \boldsymbol{G}(\boldsymbol{p}^+) = 0. \tag{S22}$$

To construct a trajectory, it is worthwhile to consider the linearized dynamics close to the equilibrium points which may be written as

$$\dot{\boldsymbol{p}} = \left.\frac{\partial \boldsymbol{G}}{\partial \boldsymbol{p}}\right|_{\boldsymbol{p}^-} \boldsymbol{p} \quad \text{ and } \quad \dot{\boldsymbol{p}} = \left.\frac{\partial \boldsymbol{G}}{\partial \boldsymbol{p}}\right|_{\boldsymbol{p}^+} \boldsymbol{p}. \tag{S23}$$

The Jacobian matrix $\partial \boldsymbol{G}/\partial \boldsymbol{p}$ represents the derivative of every component of $\boldsymbol{G}$ with respect to every component of $\boldsymbol{p}$ and is then evaluated at each of the equilibria. The solutions to the linearized systems are of the form

$$\boldsymbol{p}(z) = \widehat{\boldsymbol{p}}\, e^{\lambda z}. \tag{S24}$$

Inserting this ansatz into eq. (S23) yields eigenvalue problems for the Jacobians

$$\left.\frac{\partial \boldsymbol{G}}{\partial \boldsymbol{p}}\right|_{\boldsymbol{p}^{\pm}} \widehat{\boldsymbol{p}} = \lambda \widehat{\boldsymbol{p}} \tag{S25}$$

with the real parts of the eigenvalues being the exponential growth/decay rates and the imaginary parts representing frequencies. The five eigenvalues for $\boldsymbol{p}^{+}$ read:

$$\lambda_{1,2} = 0 \tag{S26}$$

$$\lambda_{3,4} = -\frac{c}{2D}\left(1 \pm \sqrt{1 + 4\frac{n^0 D k_2}{c^2}}\right) \tag{S27}$$

$$\lambda_5 = \frac{k_4}{c} \tag{S28}$$

Because of the two eigenvalues being zero, the point $\boldsymbol{p}^{+}$ is said to be non-hyperbolic; it has a center manifold. To further analyze the solution structure, we would apply center manifold theory reducing the dynamics to a smaller subspace which is beyond the scope of this paper.

The five eigenvalues for $\boldsymbol{p}^{-}$ are:

$$\lambda_1 = \frac{k_4}{c} \tag{S29}$$

$$\lambda_2 = \frac{n^0 \alpha k_2}{c} \tag{S30}$$

$$\lambda_3 = \frac{n^0 \alpha k_3}{c} \tag{S31}$$

$$\lambda_{4,5} = -\frac{c}{2D}\left(1 \pm \sqrt{1 + 16 n^0 D \frac{\alpha k_{-1} + (1-\alpha) k_1}{c^2}}\right) \tag{S32}$$

We note that all eigenvalues are real and cannot become zero. Therefore, the equilibrium point is hyperbolic and the form of the trajectory close to the equilibrium is completely determined by the linearized system due to the Hartman-Grobman theorem. We obtain a four-dimensional unstable manifold and a one-dimensional stable manifold. The trajectory must be contained in the unstable manifold. By close inspection of the corresponding $\widehat{\boldsymbol{p}}$ eigenvectors, none of the eigenvalues can be ruled out. We cannot find a bifurcation or restriction on c and hence the solution close to $\boldsymbol{p}^{-}$ is composed by a superposition of all four unstable eigenvectors. In contrast to the well-known Fisher's equation and successors thereof, we only find the trivial lower bound, $c > 0$ for the frontal velocity but other than this there are no restrictions originating from standard analysis of dynamical systems.

S12. Numerical solution of the reaction-diffusion equations

This section is dedicated to the numerical solution of the reaction-diffusion system. It is discretized using operator splitting. The reader finds detailed discussions about this and the methods being applied in the following in the book of Leveque[24] and references therein. First, the time tendencies due to diffusion are solved separately from the reaction part.

The Laplace operator is approximated by a second-order finite central difference. We integrate in time making use of the Euler backward method which is first order and implicit. Implicit methods have the great advantage to be applicable for stiff systems having multiple slow and fast time scales. Note that the system under consideration turns out to be stiff due to the vast differences in the kinetic rate constants (cf. Table 1). For the then necessary matrix inversion the tridiagonal matrix algorithm is employed. Second, using the preliminary result of only diffusion, the numerical scheme is completed by the reaction part which is also approximated with the backward Euler method. Since the reaction terms are nonlinear, the actual time step is performed in terms of Newton's method. Time and space are discretized equidistantly,

$$x_j = j\Delta x \tag{S33}$$

$$t_m = m\Delta t \tag{S34}$$

with m and j integers. The resulting numerical scheme is a two-stage algorithm

$$\text{Stage 1: } \frac{\hat{\boldsymbol{n}}_j - \boldsymbol{n}_j^m}{\Delta \mathrm{t}} = \mathbf{D}\,\frac{\hat{\boldsymbol{n}}_{j+1} - 2\hat{\boldsymbol{n}}_j + \hat{\boldsymbol{n}}_{j-1}}{\Delta x^2} \tag{S35}$$

$$\text{Stage 2: } \frac{\boldsymbol{n}_j^{m+1} - \hat{\boldsymbol{n}}_j}{\Delta \mathrm{t}} = \boldsymbol{F}\left(\boldsymbol{n}_j^{m+1}\right). \tag{S36}$$

We use Neumann boundary conditions: the gradients of the concentrations are set to zero at the boundaries. The initial condition is given by a prescribed, a-priori estimated front specified by

$$\boldsymbol{n}(x,0) = g(x)\boldsymbol{n}_- + \big(1 - g(x)\big)\boldsymbol{n}_+ \tag{S37}$$

$$g(x) = e^{\frac{-x^2}{\sigma^2}}. \tag{S38}$$

Thus, the initial condition at the left boundary is $\boldsymbol{n}_-$ and fulfills the Neumann condition exactly. And it is $\boldsymbol{n}_+$ at the right boundary and fulfills the Neumann condition approximately given that σ is sufficiently small. The sensitivity to different values of σ was tested, specifically with regard to the frontal velocity. We found that altering σ influences the period after which a frontal solution settles but not the final frontal velocity.

S13. LEEM and LEED observation of reaction waves

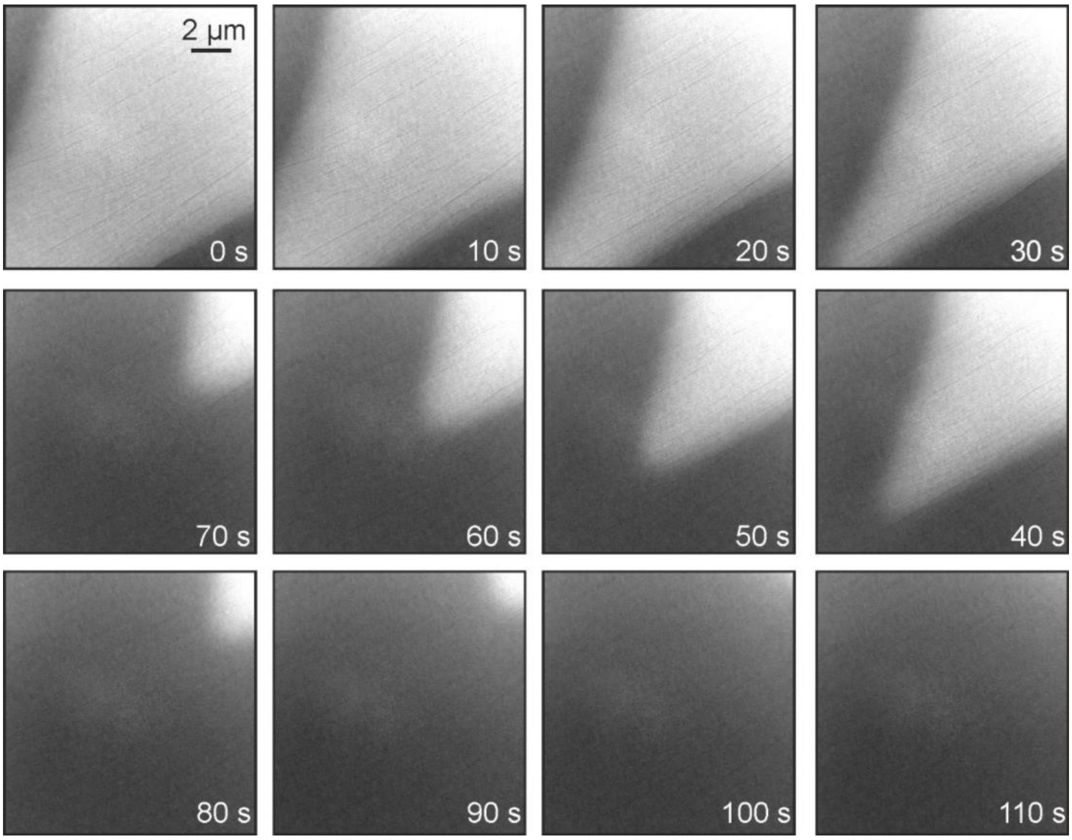

Figure S8. LEEM snapshots for the water formation reaction confined under a crystalline SiO_2 BL supported on Ru(0001). Snapshots were collected at 550 K in 1×10^{-6} mbar H_2. Kinetic electron energy is 10 eV.

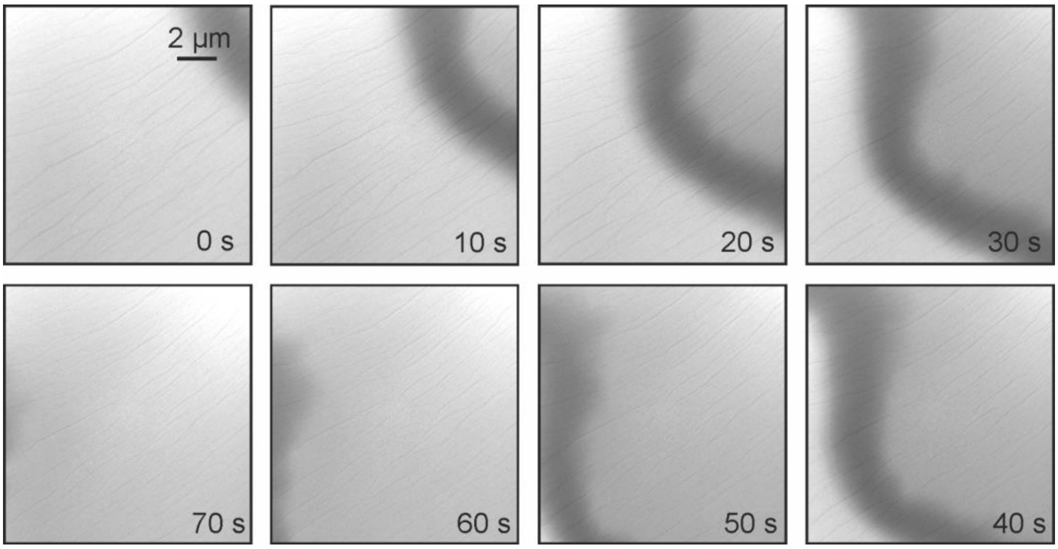

Figure S9. LEEM snapshots for the water formation reaction on Ru(0001). Snapshots were collected at 425 K in 1×10^{-6} mbar H_2. Kinetic electron energy is 12 eV.

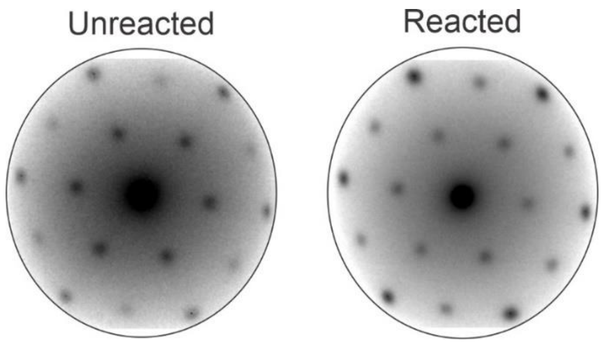

Figure S10. μ-spot LEED patterns collected on the two sides of the reaction front, as indicated. The patterns were measured at room temperature and with an electron energy of 42 eV.

S14. Transition state structures, kinetic constants and phase diagram

Table S1. Kinetic constants (k_i) obtained at different temperatures for the different reaction steps presented in equations 1-4 in the main text, as well as that for H diffusion on Ru(0001) and the two SiO_2/Ru(0001) scenarios.

System	T [K]	k_{-1} [m²/s]	k_1 [m²/s]	k_2 [m²/s]	k_3 [m²/s]	k_4 [1/s]	D_H [m²/s]
bare	398	5.2×10^{-07}	7.9×10^{-14}	4.1×10^{-19}	7.0×10^{-10}	$8.3\times10^{+12}$	2.2×10^{-09}
	423	5.7×10^{-07}	2.9×10^{-14}	2.5×10^{-18}	1.2×10^{-09}	$8.8\times10^{+12}$	2.7×10^{-09}
	448	6.2×10^{-07}	1.2×10^{-14}	1.2×10^{-17}	2.1×10^{-09}	$9.3\times10^{+12}$	3.6×10^{-09}
	473	6.7×10^{-07}	5.4×10^{-15}	5.2×10^{-17}	3.3×10^{-09}	$9.9\times10^{+12}$	4.5×10^{-09}
	500	7.2×10^{-07}	2.5×10^{-15}	2.1×10^{-16}	5.1×10^{-09}	$1.0\times10^{+13}$	5.6×10^{-09}
Constrained SiO_2 BL	500	2.9×10^{-13}	3.5×10^{-22}	1.0×10^{-16}	1.3×10^{-13}	$1.8\times10^{+08}$	1.9×10^{-09}
	543	1.2×10^{-12}	4.7×10^{-22}	7.6×10^{-16}	5.8×10^{-13}	$3.5\times10^{+08}$	3.4×10^{-09}
	576	3.0×10^{-12}	5.7×10^{-22}	2.8×10^{-15}	1.6×10^{-12}	$5.3\times10^{+08}$	4.7×10^{-09}
	613	7.5×10^{-12}	7.0×10^{-22}	1.1×10^{-14}	4.2×10^{-12}	$8.1\times10^{+08}$	6.9×10^{-09}
	665	2.3×10^{-11}	8.9×10^{-22}	5.2×10^{-14}	1.4×10^{-11}	$1.3\times10^{+09}$	1.1×10^{-08}
Optimized SiO_2 BL	500	2.6×10^{-13}	4.5×10^{-23}	2.3×10^{-16}	1.2×10^{-08}	$3.8\times10^{+00}$	1.9×10^{-09}
	543	1.0×10^{-12}	7.1×10^{-23}	1.5×10^{-15}	2.0×10^{-08}	$3.5\times10^{+01}$	3.4×10^{-09}
	576	2.7×10^{-12}	9.8×10^{-23}	5.6×10^{-15}	2.8×10^{-08}	$1.5\times10^{+02}$	4.7×10^{-09}
	613	6.7×10^{-12}	1.3×10^{-22}	2.0×10^{-14}	3.9×10^{-08}	$6.7\times10^{+02}$	6.9×10^{-09}
	665	2.1×10^{-11}	2.0×10^{-22}	9.5×10^{-14}	6.0×10^{-08}	$4.1\times10^{+03}$	1.1×10^{-08}

Table S2. Summary of kinetic constant and diffusion coefficient values at 500 K used for kinetic model simulations corresponding to the tuned case. In comparison to untuned case in table S1, the k_{-1} and k_1 values are factor 1000 larger.

	k_{-1} [m²/s]	k_1 [m²/s]	k_2 [m²/s]	k_3 [m²/s]	k_4 [1/s]	D [m²/s]
Bare	7.2×10^{-04}	2.5×10^{-12}	2.1×10^{-16}	5.1×10^{-09}	$1.0\times10^{+13}$	5.9×10^{-09}
Constrained SiO_2 BL	2.9×10^{-10}	3.5×10^{-19}	1.0×10^{-16}	1.3×10^{-13}	$1.8\times10^{+08}$	1.9×10^{-09}
Optimized SiO_2 BL	2.6×10^{-10}	4.5×10^{-20}	2.3×10^{-16}	1.2×10^{-08}	$3.8\times10^{+00}$	1.9×10^{-09}

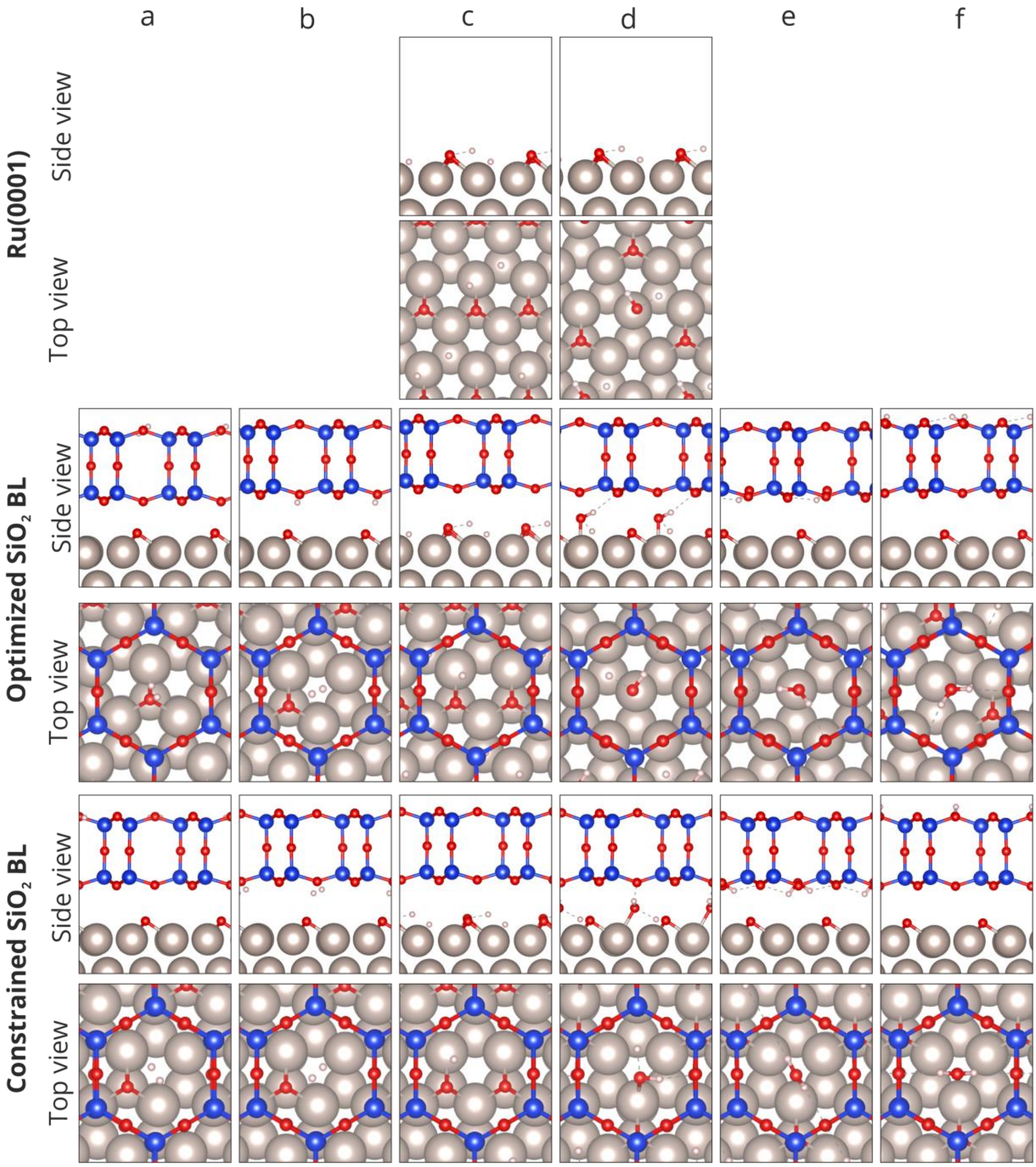

Figure S11. Transition state structures resulting from the DFT calculations and reported in the energy diagram plot of Fig. 3 (main text) for the non-confined and the different scenarios in confinement. Letters a-f in the top row correspond to the different positions in the energy diagram of Fig. 3.

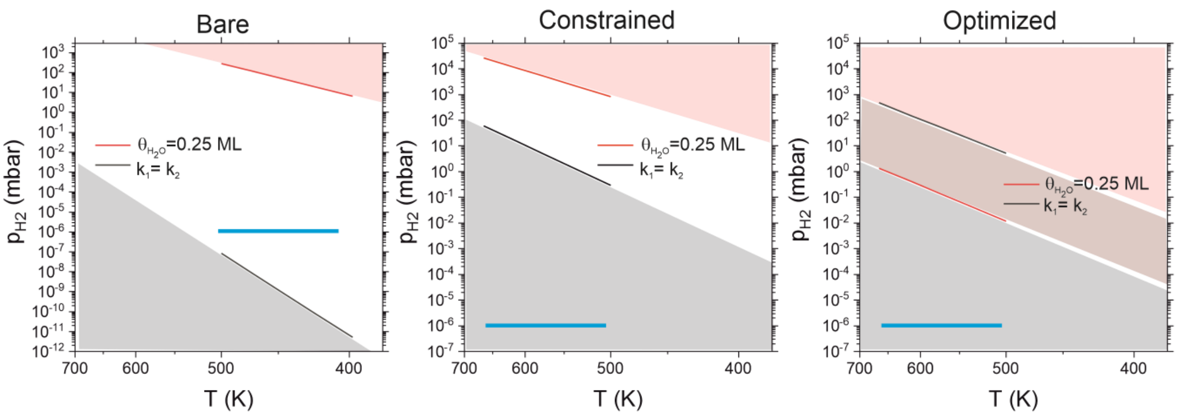

Figure S12. Phase diagrams for the different scenarios, as indicated, constructed from the dependance of the three most important steps of the reaction: hydrogen adsorption (k_1), hydroxyl formation (k_2) and water desorption k_4. Grey area: reaction kinetics limited by the adsorption of H_2 ($k_1 < k_2$). White area: OH formation is the step limiting the kinetics ($k_2 < k_1$). Red area: Kinetics is limited by the low desorption of H_2O ($\theta_{H_2O}> 0.25$ ML). In the case of the optimized SiO_2 BL scenario red-grey area corresponds to a parameter space where the kinetic limitation is due to both, H_2 adsorption and H_2O desorption. Blue line corresponds to the conditions used for our experiments on the bare and silica BL covered Ru(0001) surface.

References

1. Fink, R.; Weiss, M. R.; Umbach, E.; Preikszas, D.; Rose, H.; Spehr, R.; Hartel, P.; Engel, W.; Degenhardt, R.; Wichtendahl, R.; Kuhlenbeck, H.; Erlebach, W.; Ihmann, K.; Schlögl, R.; Freund, H. J.; Bradshaw, A. M.; Lilienkamp, G.; Schmidt, T.; Bauer, E.; Benner, G., SMART: A planned ultrahigh-resolution spectromicroscope for BESSY II. *J Elect Spect and Relat Phenomena* **1997,** *84* (1-3), 231-250.
2. Schmidt, T.; Marchetto, H.; Lévesque, P. L.; Groh, U.; Maier, F.; Preikszas, D.; Hartel, P.; Spehr, R.; Lilienkamp, G.; Engel, W.; Fink, R.; Bauer, E.; Rose, H.; Umbach, E.; Freund, H. J., Double aberration correction in a low-energy electron microscope. *Ultramicroscopy* **2010,** *110* (11), 1358-61.
3. Schmidt, T.; Sala, A.; Marchetto, H.; Umbach, E.; Freund, H. J., First experimental proof for aberration correction in XPEEM: resolution, transmission enhancement, and limitation by space charge effects. *Ultramicroscopy* **2013,** *126*, 23-32.
4. Klemm, H. W.; Prieto, M. J.; Peschel, G.; Fuhrich, A.; Madej, E.; Xiong, F.; Menzel, D.; Schmidt, T.; Freund, H.-J., Formation and Evolution of Ultrathin Silica Polymorphs on Ru(0001) Studied with Combined in Situ, Real-Time Methods. *J Phys Chem C* **2018**.
5. Gsell, M.; Stichler, M.; Jakob, P.; Menzel, D., Formation and geometry of a high-coverage oxygen adlayer on Ru(001), the p(2 x 2)-30 phase. *Israel J Chem* **1998,** *38* (4), 339-348.
6. Kostov, K. L.; Gsell, M.; Jakob, P.; Moritz, T.; Widdra, W.; Menzel, D., Observation of a novel high density 3O(2 × 2) structure on Ru(001). *Surf Sci* **1997,** *394* (1), L138-L144.
7. Kohn, W.; Sham, L. J., Self-Consistent Equations Including Exchange and Correlation Effects. *Phys Rev* **1965,** *140* (4A), A1133-A1138.
8. Perdew, J. P.; Burke, K.; Ernzerhof, M., Generalized Gradient Approximation Made Simple. *Phys Rev Lett* **1996,** *77* (18), 3865-3868.
9. Grimme, S., Semiempirical GGA-type density functional constructed with a long-range dispersion correction. *J Comp Chem* **2006,** *27* (15), 1787-1799.
10. Hafner, J.; Kresse, G., The Vienna AB-Initio Simulation Program VASP: An Efficient and Versatile Tool for Studying the Structural, Dynamic, and Electronic Properties of Materials. In *Properties of*

Complex Inorganic Solids, Gonis, A.; Meike, A.; Turchi, P. E. A., Eds. Springer US: Boston, MA, 1997; pp 69-82.
11. Giannozzi, P.; Baroni, S.; Bonini, N.; Calandra, M.; Car, R.; Cavazzoni, C.; Ceresoli, D.; Chiarotti, G. L.; Cococcioni, M.; Dabo, I.; Dal Corso, A.; de Gironcoli, S.; Fabris, S.; Fratesi, G.; Gebauer, R.; Gerstmann, U.; Gougoussis, C.; Kokalj, A.; Lazzeri, M.; Martin-Samos, L.; Marzari, N.; Mauri, F.; Mazzarello, R.; Paolini, S.; Pasquarello, A.; Paulatto, L.; Sbraccia, C.; Scandolo, S.; Sclauzero, G.; Seitsonen, A. P.; Smogunov, A.; Umari, P.; Wentzcovitch, R. M., QUANTUM ESPRESSO: a modular and open-source software project for quantum simulations of materials. *J Phys: Condensed Matter* **2009,** *21* (39), 395502.
12. Monkhorst, H. J.; Pack, J. D., Special points for Brillouin-zone integrations. *Phys Rev B* **1976,** *13* (12), 5188-5192.
13. Jónsson, H.; Mills, G.; Jacobsen, K. W., Nudged elastic band method for finding minimum energy paths of transitions. In *Classical and Quantum Dynamics in Condensed Phase Simulations*, pp 385-404.
14. Henkelman, G.; Jónsson, H., A dimer method for finding saddle points on high dimensional potential surfaces using only first derivatives. *J Chem Phys* **1999,** *111* (15), 7010-7022.
15. Arblaster, J. W., Crystallographic properties of ruthenium. *Platinum Metals Review* **2013,** *57* (2), 127-136.
16. Feulner, P.; Menzel, D., The adsorption of hydrogen on ruthenium (001): Adsorption states, dipole moments and kinetics of adsorption and desorption. *Surf Sci* **1985,** *154* (2), 465-488.
17. Herron, J. A.; Tonelli, S.; Mavrikakis, M., Atomic and molecular adsorption on Ru(0001). *Surf Sci* **2013,** *614*, 64-74.
18. Stampfl, C.; Scheffler, M., Theoretical study of O adlayers on Ru(0001). *Phys Rev B* **1996,** *54* (4), 2868-2872.
19. Yang, B.; Kaden, W. E.; Yu, X.; Boscoboinik, J. A.; Martynova, Y.; Lichtenstein, L.; Heyde, M.; Sterrer, M.; Wlodarczyk, R.; Sierka, M.; Sauer, J.; Shaikhutdinov, S.; Freund, H. J., Thin silica films on Ru(0001): monolayer, bilayer and three-dimensional networks of [SiO4] tetrahedra. *Phys Chem Chem Phys* **2012,** *14* (32), 11344-11351.
20. Michaelides, A.; Alavi, A.; King, D. A., Different Surface Chemistries of Water on Ru{0001}: From Monomer Adsorption to Partially Dissociated Bilayers. *J Am Chem Soc* **2003,** *125* (9), 2746-2755.
21. Beyer, M. K., Lehrbuch der Physikalischen Chemie. 6th Edition, with Workbook (in German). By Gerd Wedler and Hans-Joachim Freund. *Angew Chem Int Ed* **2013,** *52* (14), 3805-3805.
22. Marin, G. B.; Yablonsky, G. S.; Constales, D., *Kinetics of chemical reactions: Decoding complexity*. 2nd ed.; Wiley-VCH Verlag GmbH & Co. KGaA: Weinheim, Germany, 2019.
23. Sachs, C.; Hildebrand, M.; Völkening, S.; Wintterlin, J.; Ertl, G., Reaction fronts in the oxidation of hydrogen on Pt(111): Scanning tunneling microscopy experiments and reaction–diffusion modeling. *J Chem Phys* **2002,** *116* (13), 5759-5773.
24. Leveque, R. J., *Finite Volume Methods for Hyperbolic Problems*. Cambridge University Press: 2002; p 580.
25. Lambert, J. D., *Numerical Methods for Ordinary Differential Systems*. Wiley: 1992.
